# Warmer temperature during asexual reproduction induce methylome, transcriptomic, and lasting phenotypic changes in *Fragaria vesca* ecotypes

**DOI:** 10.1093/hr/uhad156

**Published:** 2023-07-31

**Authors:** YuPeng Zhang(张宇鹏), Guangxun Fan, Tuomas Toivainen, Torstein Tengs, Igor Yakovlev, Paal Krokene, Timo Hytönen, Carl Gunnar Fossdal, Paul E. Grini

**Affiliations:** EVOGENE, Department of Biosciences, University of Oslo, 0313 Oslo, Norway; Department of Molecular Plant Biology, Norwegian Institute of Bioeconomy Research, 1431 Ås, Norway; Department of Agricultural Sciences, Viikki Plant Science Centre, University of Helsinki, 00014 Helsinki, Finland; Department of Agricultural Sciences, Viikki Plant Science Centre, University of Helsinki, 00014 Helsinki, Finland; Department of Molecular Plant Biology, Norwegian Institute of Bioeconomy Research, 1431 Ås, Norway; Department of Molecular Plant Biology, Norwegian Institute of Bioeconomy Research, 1431 Ås, Norway; Department of Molecular Plant Biology, Norwegian Institute of Bioeconomy Research, 1431 Ås, Norway; Department of Agricultural Sciences, Viikki Plant Science Centre, University of Helsinki, 00014 Helsinki, Finland; Department of Molecular Plant Biology, Norwegian Institute of Bioeconomy Research, 1431 Ås, Norway; EVOGENE, Department of Biosciences, University of Oslo, 0313 Oslo, Norway

## Abstract

Plants must adapt with increasing speed to global warming to maintain their fitness. One rapid adaptation mechanism is epigenetic memory, which may provide organisms sufficient time to adapt to climate change. We studied how the perennial *Fragaria vesca* adapted to warmer temperatures (28°C vs. 18°C) over three asexual generations. Differences in flowering time, stolon number, and petiole length were induced by warmer temperature in one or more ecotypes after three asexual generations and persisted in a common garden environment. Induced methylome changes differed between the four ecotypes from Norway, Iceland, Italy, and Spain, but shared methylome responses were also identified. Most differentially methylated regions (DMRs) occurred in the CHG context, and most CHG and CHH DMRs were hypermethylated at the warmer temperature. In eight CHG DMR peaks, a highly similar methylation pattern could be observed between ecotypes. On average, 13% of the differentially methylated genes between ecotypes also showed a temperature-induced change in gene expression. We observed ecotype-specific methylation and expression patterns for genes related to gibberellin metabolism, flowering time, and epigenetic mechanisms. Furthermore, we observed a negative correlation with gene expression when repetitive elements were found near (±2 kb) or inside genes. In conclusion, lasting phenotypic changes indicative of an epigenetic memory were induced by warmer temperature and were accompanied by changes in DNA methylation patterns. Both shared methylation patterns and transcriptome differences between *F. vesca* accessions were observed, indicating that DNA methylation may be involved in both general and ecotype-specific phenotypic variation.

## Introduction

Plants growing in extreme climates are under particularly strong natural selection to synchronize their growth with the environment. In addition to allelic variation, epigenetic modifications provide a mechanism for short-term adaptation to environmental change and preservation of genetic variation. By increasing phenotypic variation, epigenetic modifications influence many plant traits, including flowering time [1–3], flower color [4, 5], vegetative growth [6], and kernel color [7, 8].

Epigenetic mechanisms, such as DNA methylation, histone modification, and small RNA-dependent pathways, modify the accessibility of DNA in chromatin and thus modulate gene expression and alter the plant phenotype. In the context of environmental change, direct changes in DNA methylation due to environmental impacts can be triggered by changes in various biotic or abiotic parameters, including increased temperature [9–11, 106]. Canonical DNA methylation refers to 5-methylcytosine, where cytosine bases of the DNA are modified by the addition of a methyl group [12]. Canonical DNA methylation occurs in the CGN, CHG, and CHH contexts (where N can be any base and H can be A, C, or T). While they are rare in mammals and other organisms, the CHG and CHH contexts are common in plants [13, 14].

Plants have three main classes of methyltransferases, which transfer and covalently attach methyl groups onto DNA [15, 16]. The METHYLTRANSFERASE 1 (MET1), CHROMOMETHYLASE 3 (CMT3), and DOMAINS REARRANGED METHYLTRANSFERASE 1 and 2 (DRM1/DRM2) have different roles in DNA methylation [17–19]. For example, CHG and CHH have been identified as regulators of transposon silencing, while CGN may be associated with gene–body regions and poly-A tail length in plants [20–22]. MET1 maintains DNA methylation in the CGN context, CMT3 is in charge of maintenance of CHG methylation, while DRM maintains CHH methylation as a *de novo* DNA methylation methyltransferase [17–19, 23].

Epigenetic regulators can also act in concert. For example, the concerted action of DNA methyltransferases CMT2 and CMT3 plays an important role in histone 3 lysine 9 (H3K9) methylation. The KRYPTONITE (KYP) protein reads both CHG and CHH DNA methylation contexts via SET and RING-associated domains and performs histone methylation on nearby chromatin [23–26]. Such CHG and CHH DNA methylation depends on CMT2 and CMT3 as well as KYP and its homologs SUPPRESSOR OF VARIEGATION 3–9 HOMOLOGUE 5 (SUVH5) and SUVH6 [24, 27]. RELATIVE OF EARLY FLOWERING 6, a Jumonji domain-containing histone demethylase, recognizes a specific CHG-containing motif in hypomethylated regions, thus causing targeted histone demethylation [28, 29].

Stable epigenetic marks can be inherited to the next generation. The classical example is toadflax (*Linaria vulgaris*), where a natural epimutation of *Lcyc*, a homolog of the *cycloidea* gene, changes flower symmetry [30, 31]. When an Arabidopsis lineage with a hypomethylated genome is crossed with a normal wild type, massive methylation and expression changes occur in the next generation [32]. More intriguingly, organ-specific methylation markers are inherited by asexual offspring and can be maintained even through the following meiosis [33]. However, also epialleles induced by abiotic and biotic environments can be inherited. When *Arabidopsis thaliana* (*Arabidopsis*) faces high osmotic stress, an epigenetic memory is induced that can be passed onto the next generation, mainly by female gametes [10].

The most detailed epigenetic mechanism related to temperature in angiosperms is vernalization. Vernalization is the induction of the flowering process by exposure to the cold of winter, or to artificial chill hours treatment [34]. Epigenetic memory to warming conditions have earlier been described for epitypes in the gymnosperm Norway spruce [9]. Adaptive traits such as the timing of bud burst and bud set were affected epigenetically in a lasting manner. This was observed in trees growing under identical conditions in the field that had earlier experienced 28°C versus 18°C only during embryogenesis [35–41]. This may be an adaptive mechanism that provides plants the plasticity needed to cope with climatic changes acting faster than classical mendelian selection [35]. Here we wanted to explore whether the angiosperm *Fragaria vesca* has a similar epigenetic memory response to warmer conditions.

We used *F. vesca* (2*n* = 2*x* = 14) to investigate the presence of a temperature-induced epigenetic memory in angiosperm plants [42, 43]. *F. vesca* has a genome size of 219 Mb distributed on seven chromosomes with an estimated gene number of 34 007 protein coding genes [44, 45]. It is distributed throughout Eurasia and is an ancestor of commercial strawberries. It has a relatively short generation cycle of both asexual and sexual reproduction, making it an ideal system to study climatic responses in perennial plants. Using four *F. vesca* ecotypes, we tested for lasting phenotypic impacts of different temperature conditions (28°C and 18°C) for up to three generations of asexual propagation. The choice of the 18°C as the control condition temperature (which is also the common garden condition used) is based on the consideration that this is a normal growth condition for *F. vesca*. At this temperature under controlled conditions, *F. vesca* can be induced for flowering using short day (SD) treatment, while only a 3°C increase to 21°C tends to inhibit SD-induced flowering [46]. DNA methylation was quantified in leaf tissues, and differentially methylated regions (DMRs) (region consisted with differentially methylated cytosines), genes (DMGs, gene with DMRs), repetitive elements (REs) (and transposons), and other genic features associated with DMRs and differentially expressed genes (DEGs, changed transcripts levels) were compared. We demonstrate that *F. vesca* has a temperature-induced epigenetic memory and that phenotypic changes induced by warmer temperatures are accompanied by changes in DNA methylation patterns. By shedding light on plant adaptation to warmer temperature conditions (not stress), our study offers valuable insights for developing strategies to mitigate the impacts of climate change on plants. The identified findings hold potential for targeting epigenetically regulated genes, enabling the enhancement of crop adaptation to warmer climates through the application of epigenome editing techniques.

## Results

### Warmer temperatures induce phenotypic variation

**Figure 1:**
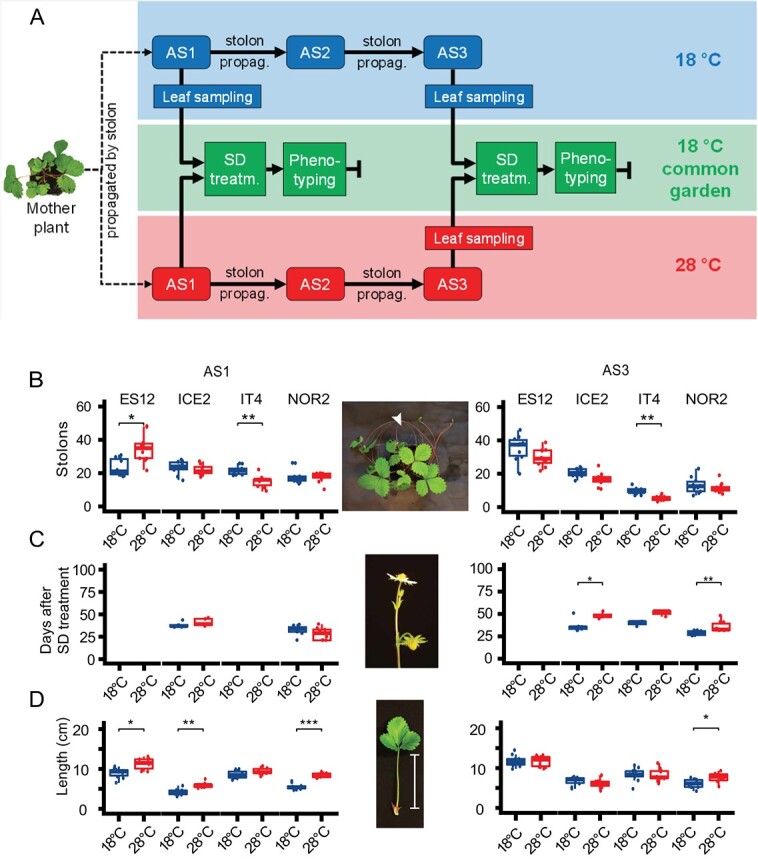
Experimental setup and phenotypic responses under common garden-conditions in *Fragaria vesca* plants after propagation at different temperatures for up to three asexual generations. **(A)** Experimental setup: plants were propagated for three asexual generations (AS1–3) through stolon formation at 18°C (blue boxes) or 28°C (red boxes). Leaf samples for DNA and RNA sequencing were collected from AS1 plants (at 18°C only) and AS3 plants (at both temperatures). Phenotypic observations of AS1 and AS3 plants were made under common garden-conditions following short-day (SD) treatment (green boxes). **(B-D)** Total number of stolons **(B)**, flowering time **(C)**, and petiole length **(D)** in the ES12, ICE2, IT4, and NOR2 ecotypes. Plants were propagated at 18°C or 28°C and phenotypes were scored under common garden-conditions after flower-inducing short-day (SD) treatment. Flowering time was measured as days from the start of SD treatment until the first flower had opened completely. Flowering time could not be determined in ES12 plants since these could not be induced to flower. Arrowhead in the upper image indicates stolon; the bracket in the lower image indicates petiole length. Brackets and asterisks indicate significant differences from Wilcoxon tests: ^*^ 0.01 ≤ p < 0.05; ^**^ 0.001 ≤ p < 0.01; ^***^ 0.0001 ≤ p < 0.001. § p = 0.06. Error bars show 95% confidence intervals. Box plots also show median values (n = 10) and the interquartile range (difference between the 75th and 25th percentiles).

We tested whether a substantial difference in temperature experienced during asexual propagation generates heritable epigenetic alterations across three asexual generations in four European *F. vesca* ecotypes (Supplementary Figure S1). Clonally propagated plants originating from one individual per ecotype were grown at 18°C and 28°C for three asexual generations (Figure 1A). To uncover any long-term phenotypic changes indicative of a temperature-induced epigenetic memory, we examined three phenotypic parameters under common garden-conditions after one and three asexual generations (AS1, AS3). Flowering time was chosen as this parameter has been impacted epigenetically in previous studies [47–49] and directly relate to reproductive fitness. Stolon number was chosen as it is often studied in combination with flowering as opposite patterns are often observed, i.e., how much effort is put into sexual (flowering) versus asexual (stolon) based reproduction [46, 48]. As a general proxy for plant growth, petiole length was investigated [46].

The Spanish ES12 ecotype produced significantly more stolons in the common garden after propagation at 28°C than at 18°C in AS1, but not in AS3 (Figure 1B; Supplementary Datasheet S1). The Italian IT4 ecotype, on the other hand, produced significantly fewer stolons at 28°C both in AS1 and AS3 (Figure 1B, Supplementary Datasheet S1, 0.05 > *p* > 0.001). Notably, IT4 plants propagated at 28°C stopped generating stolons from week 11 onwards in AS3 (Supplementary Figure S2, Supplementary Datasheet S1). Stolon production was not altered significantly by temperature in the Icelandic ICE2 and Norwegian NOR2 ecotypes (Figure 1B, Supplementary Figure S2, Supplementary Datasheet S1).

Temperature conditions affected flowering time considerably (Figure 1C, Supplementary Datasheet S1). In AS1, only ICE2 and NOR2 could be induced to flower and there were no significant effects of temperature on flowering time. In AS3, however, flowering occurred significantly later in ICE2 and NOR2 plants propagated at 28°C than at 18°C (Figure 1C, Supplementary Datasheet S1, 0.05 > *p* > 0.001). Three out of 10 IT4 plants flowered in AS3 and there were no significant differences between temperatures (Figure 1C, Supplementary Datasheet S1). Interestingly, flowering time change and stolon number change were never observed concomitantly within the same ecotype (Fig. 1), highlighting that these two phenotypic parameters represent two reproductive modes that are antagonistic and may be adapted differently in the ecotypes. The ES12 ecotype did not flower at all under our experimental conditions, indicating that it has different environmental requirements for flowering.

Petiole length in the common garden was significantly longer in AS1 in ES12, ICE2, and NOR2 plants propagated at 28°C than at 18°C (Figure 1D, Supplementary Datasheet S1; 0.05 > *p* > 0.0001). Differences in petiole length disappeared in AS3 for ES12 and ICE2 but persisted for NOR2 (Figure 1D, Supplementary Datasheet S1, 0.05 > *p* > 0.0001). In summary, we observed statistically significant differences between temperature conditions for all phenotypic traits investigated under common garden conditions, indicating the presence of a memory effect of the temperature experienced during asexual propagation.

### Global differences in DNA methylation between ecotypes

We sequenced bisulfite-treated genomic DNA samples (>20× genome coverage) from young leaves of all four *F. vesca* ecotypes propagated at 18°C and 28°C (Supplementary Figure S1, Supplementary Datasheet S2). Due to the different roles of the DNA methylation contexts, we compared global cytosine methylation in the CGN (~55% methylated), CHG (~30%), and CHH (~6%) methylation contexts at 18°C using AS3 plants (Supplementary Figure S3, Supplementary Datasheet S2). The most densely methylated regions on the different chromosomes corresponded with centromeric and pericentromeric heterochromatin (Supplementary Figure S4) [50]. Individual *F. vesca* chromosomes showed distinct methylation patterns and these patterns were similar in all four ecotypes (Supplementary Figure S3,S4). ICE2 was overall hypermethylated compared to all other ecotypes at both a single-chromosome and whole-genome level (Supplementary Figure S5, Supplementary Datasheet S3). We also analyzed the importance of specific trinucleotide contexts of methylated CGN, CHG, and CHH sites on a chromosomal scale. Notably, CCG was less methylated than other sites (Supplementary Figures S6,S7 andS8), as reported previously [51].

Linear regression analysis revealed an inverse relationship between protein-coding gene density and chromosome methylation levels (Supplementary Figure S9). We found a positive correlation between DNA methylation and REs, as well as pseudogenes (Supplementary Datasheet S4). Linear regression models considering all genomic features explained ~30% to 55% of the methylation (50% of CGN, 55% of CHG, 30% CHG) (Supplementary Datasheet S4).

### Global methylation changes are induced by temperature conditions

**Figure 2:**
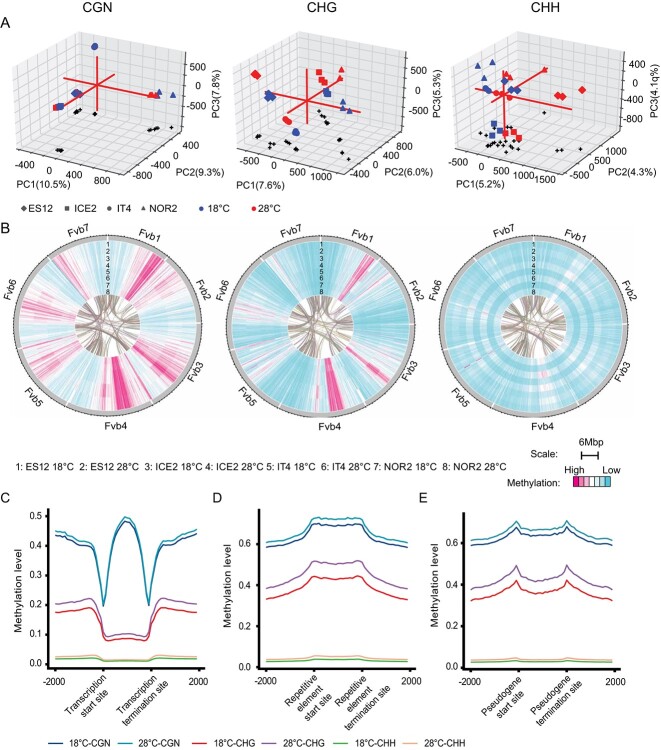
DNA methylation landscape in leaves of four *Fragaria vesca* ecotypes propagated for three asexual generations at 18°C or 28°C. Ecotypes are from Spain (ES12), Iceland (ICE2), Italy (IT4), and Norway (NOR2). All methylation data show the CGN, CHG, and CHH methylation contexts (where N can be any base and H can be any base other than G). Three biological replicates were used for each ecotype and temperature combination. **(A)** Principal component analysis of methylation in each ecotype grown at 18°C (blue symbols) and 28°C (red symbols). Black crosses show projections of the data points on the xy-plane. Red lines indicate the x, y and z axes. **(B)** Methylation patterns along all seven *F. vesca* chromosomes for all ecotypes and both temperatures, using a 50 kb window. Lanes 1–8 show methylation levels for each ecotype-temperature combination. Connecting lines in the middle indicate coding regions (CDS) regions that showed synteny. **(C-E)** Methylation level along protein coding genes **(C)**, REs **(D)**, and pseudogenes **(E)** in the NOR2 ecotype. Each genomic feature and regions 2 kb up- and downstream to these were divided into 20 pieces to calculate methylation levels (methylated reads/total reads). Colored lines show different combinations of temperature and methylation contexts.

Principal Component Analysis (PCA) of methylation profiles in plants propagated at 18 or 28°C demonstrated little variability between biological replicates within ecotypes. However, PCA revealed differences in methylation patterns between ecotypes propagated at 18°C versus 28°C, particularly for the CHG and CHH contexts (Figure 2A). NOR2 had the largest difference in methylation levels between the two temperature conditions, as well as the most significant phenotypic difference (Figure 1, Supplementary Figure S10, Supplementary Datasheet S5).

At a 50-kb window resolution, all ecotypes had similar CGN methylation patterns, whereas CHG and CHH methylation patterns differed (Supplementary Figure S10). NOR2 had higher methylation increases at 28°C versus 18°C for all methylation contexts and all chromosomes, with an average of ~4%, ~5%, and ~ 1% hypermethylation, respectively (Supplementary Datasheet S5). No obvious methylation temperature bias of trinucleotide contexts was observed (Supplementary Figure S11,S12, S13).

We compared methylation responses to temperature conditions between all ecotypes by plotting heat-maps of chromosomes, ecotypes, and temperature conditions for all three methylation contexts. Significant changes in hypomethylation and hypermethylation occurred along the chromosomes in all ecotypes, and the largest temperature-specific methylation increases at 28°C were observed for the CHH context (Figure 2B). For the CGN and CHG contexts the most striking hypomethylated and hypermethylated areas were largely shared between ecotypes and temperature conditions (Figure 2B).

### Methylation of genomic features differs between temperature conditions

We calculated DNA methylation patterns along genes, REs, and pseudogenes, as well as 2 kb upstream and downstream of these genomic features. We noticed that methylation levels were lowest around transcription start sites (TSS) and transcription termination sites (TTS) for genes (Figure 2C, Supplementary Figure S14) [44, 52, 106], while REs showed a plateau of hypermethylation in the RE body region (Figure 2D, Supplementary Figure S14). For pseudogenes, methylation levels peaked around start and end sites and were lower within body regions (Figure 2E, Supplementary Figure S14). Methylation levels were higher in plants propagated at 28°C versus 18°C in all genomic features, both in genic and non-genic regions (Figure 2C-E, Supplementary Figure S14). To further investigate the hypermethylation observed in plants propagated at 28°C, we compared global methylation levels in genes, REs, and pseudogenes. REs and pseudogenes were significantly hypermethylated in all methylation contexts and all ecotypes (Supplementary Figure 15, Supplementary Datasheet S6). Again, the ecotype NOR2, which had the largest phenotypic difference between temperatures (Figure 1) also had the largest and most significant difference in methylation change in all genic features (Supplementary Figure S15, Supplementary Datasheet S6).

### Regions with differential CHG and CHH methylation are generally hypermethylated

**Figure 3:**
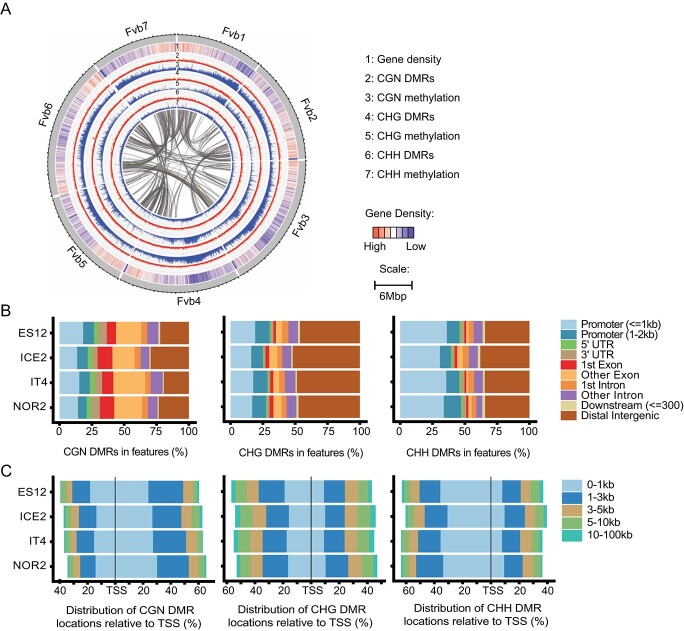
Chromosomal and genic distribution patterns of DMRs in *Fragaria vesca* leaves propagated for three asexual generations at 18°C or 28°C. **(A)** Pattern of DMRs on each *F. vesca* (Fv) chromosome in the NOR2 ecotype. Lanes 1–7 show gene density, DMR density, and methylation level (%; methylated reads/total reads, using a 50 kb window) for different methylation contexts (CGN, CHG, and CHH, where N can be any base and H can be any base other than G). Connecting lines in the middle indicate coding regions (CDS) regions that showed synteny. **(B)** Distribution of DMRs for different methylation contexts across different genomic features in four *F. vesca* ecotypes (ES12, ICE2, NOR2, and IT4). **(C)** Distribution of DMRs for different methylation contexts according to their distance (kilobase-pairs in the 5′-3′ direction) from transcription start sites (TSS) in different ecotypes. Three biological replicates were used for each ecotype and temperature combination.

We identified DMRs between ecotypes propagated at 18°C versus 28°C for three asexual generations (AS3) (Figure 3A, Supplementary Figure S16, Supplementary Datasheet S7). DMRs between plants propagated at 18°C for three vs. one asexual generation (AS3 vs. AS1) were identified as a control (Supplementary Figures S17,S18, Supplementary Datasheet S8). The total number of DMRs observed in the trans-generational control (AS3 vs. AS1) was significantly lower than that in the temperature comparison (18°C vs. 28°C; 0.05 > *p* > 0.001) (Supplementary Datasheet S9). Hypermethylated DMRs in the CHG and CHH contexts were significantly more common in the temperature comparison than in the trans-generational control (*p* < 0.008 and *p* < 0.001 for CHG and CHH, respectively). The number of DMRs for different methylation contexts varied between ecotypes, with the phenotypically most plastic ecotype NOR2 having the greatest number of DMRs for all methylation contexts. Hypermethylation was the main methylation response to warmer temperatures, as most DMRs in the CHG and CHH contexts were hypermethylated at 28°C.

### Differentially methylated regions pinpoint temperature-specific methylation peaks.

We found distinct distribution patterns of DMRs along each chromosome, with some high-density DMR regions that are shared between ecotypes, indicative of a shared epigenetic mechanism in response to a warming climate (Figure 3A, Supplementary Figure S16, Supplementary Datasheet S10). Most CHG DMRs were found in intergenic regions (Figure 3B), while CGN and CHH DMRs were primarily found in gene bodies and promoters. CHG and CHH DMRs were usually situated in the 3′ direction relative to the transcription start site, whereas CGN DMRs tended to be in the 5′ direction (Figure 3C).

To locate further similarities in DMR sequence context, we used HOMER2 analysis [53] to identify shared motifs between ecotypes. No overall enrichment was found, except a MYB transcription factor (TF) binding motif overrepresented (*p* = 1E-7 to 1E-14) in 200-bp regions adjacent to CHG DMRs (Supplementary Datasheet S11, S12). Notably, several MYBs have been indicated in response to temperature [54–57, 106], also acting in concert with epigenetic modification of the MYB binding motif [58].

Using a 50 kb window, we identified eight DMR peak regions in the CHG context on chromosomes Fvb2, Fvb3, Fvb5, and Fvb6 that were shared in all ecotypes (Supplementary Datasheet S10), and may indicate shared features that are targeted by the methylation machinery upon temperature change. These regions were adjacent to, but did not completely overlap with, methylation peaks on each chromosome and only occupied a limited region of each 50 kb window.

**Figure 4:**
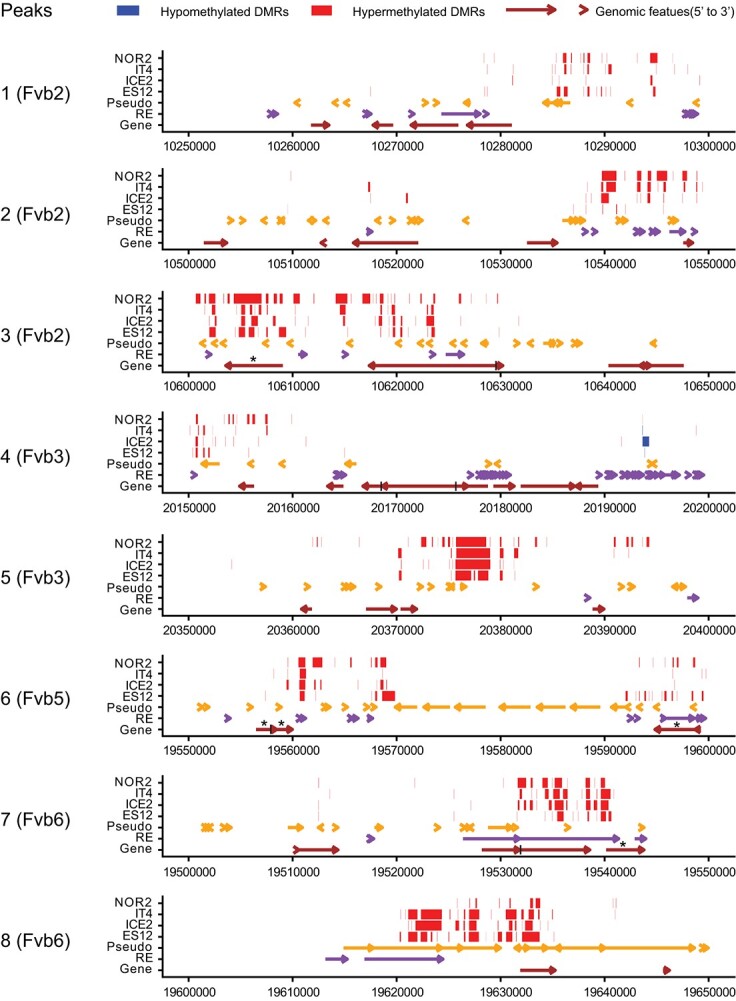
DMRs and genomic features inside DMR peaks in leaves of different *Fragaria vesca* ecotypes propagated for three asexual generations at 18°C or 28°C. Distribution of DMRs, pseudogenes, REs, and genes along eight 50 kb wide DMR peaks for the CHG methylation context (where H can be any base other than G) in four *F. vesca* (Fv) chromosomes. The x-axis indicates location along the DMR peak. Red and blue bars represent hyper- and hypo-methylated DMRs, respectively. Coloured arrows indicate pseudogenes (yellow), REs (purple), and genes (brown). Asterisks indicate predicted *TARGET OF PROTEIN FOR XKLP2* (*TPX2*) genes.

To further investigate DMR peak regions, we plotted CHG DMRs against different genomic features. We identified two to eight genes within the regions designated as DMR peak regions (Supplementary Datasheet S10). Most DMRs did not overlap with gene body regions and did not show a strong correlation with genes or other genomic features in DMR peak regions (Figure 4). However, all DRMs within peaks, except for a single region in peak 4, were hypermethylated at 28°C versus 18°C (Figure 4). Differential methylation patterns in both hypo- and hypermethylated DMR peaks were conserved between ecotypes, suggesting a common underlying methylation mechanism. A gene ontology (GO) analysis of genes located in DMR peaks identified several GO terms related to microtubule cytoskeleton organization that were enriched >250-fold (p_adj_ < 0.000002, Supplementary Datasheet S10). Five *TARGET OF PROTEIN FOR XKLP2 (TPX2)* genes (out of 21 in the whole genome) were located in three DMR peaks (peaks 3, 6, 7; Figure 4, Supplementary Datasheet S10) and were significantly enriched in peaks shared between ecotypes.

### Differentially expressed and differentially methylated genes (DEDMGs) are detected in all four ecotypes

**Figure 5:**
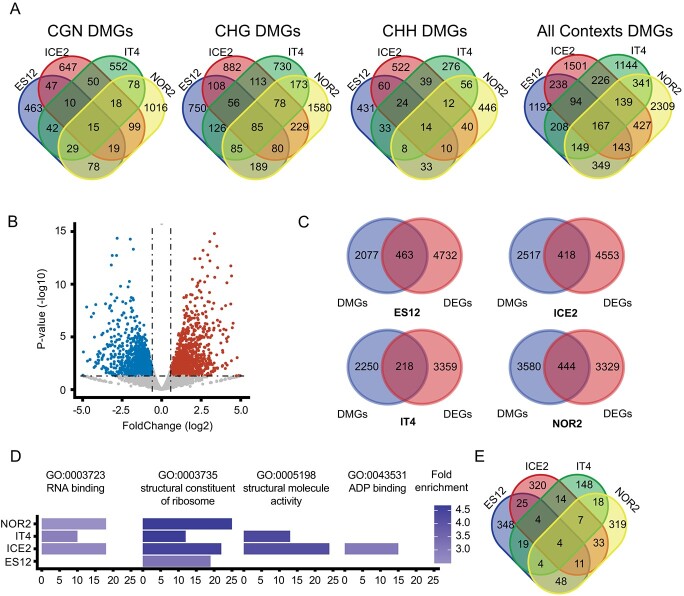
Relationship between DNA methylation and gene expression in *Fragaria vesca* ecotypes propagated for three asexual generations at 18°C or 28°C. **(A)** Venn diagrams showing numbers of differentially methylated genes (DMGs) for different ecotypes (ES12, ICE2, NOR2, IT4) and methylation contexts (CGN, CHG, CHH, where N can be any base and H can be any base other than G). **(B)** Volcano plot showing differentially expressed genes (DEGs) in NOR2 plants propagated at 28°C vs. 18°C. Blue and red dots show down- and upregulated genes, respectively. Dashed lines delineate P-value ≤0.05 and |FoldChange | ≥ 1.5 calculated from three biological replicates. **(C)** Venn diagrams showing overlap between DEGs and DMGs in the different ecotypes. **(D)** Enriched GO terms among DEDMGs in the different ecotypes. The x-axis indicates the number of DEDMGs per GO term. Color shading indicates fold enrichment at 28 vs. 18°C. **(E)** Venn diagrams showing numbers of DEDMGs in all ecotypes.

We identified differentially methylated genes (DMGs) as genes with DMRs located in promoter and/or gene body regions (Supplementary Datasheet S13). Most DMGs were methylated in the CHG context. Each ecotype had its unique set of DMGs (>1144), and only a few DMGs with the same methylation context were shared between all ecotypes (15 for CGN, 85 for CHG, and 14 for CHH) (Figure 5A, Supplementary Datasheet S13). If DMGs were defined as differentially methylated in any context, the number of shared DMGs increased to 167 (Figure 5A, Supplementary Datasheet S13).

To associate DMGs with gene expression, we profiled AS3 transcripts from all ecotypes at both temperatures (Figure 1A, Supplementary Datasheet S14). A PCA analysis demonstrated sample uniformity and clear separation between temperatures (Supplementary Figure S19). We compared DMGs to temperature-induced differentially expressed genes (DEGs) (Figure 5B, Supplementary Figure S20) and found a significant over-representation of DEDMGs in 28°C samples in three out of four ecotypes (~8–18% DEDMGs, Fisher exact test, Figure 5C, Supplementary Datasheet S14). The DEDMGs were enriched for four GO terms: RNA binding, structural component of the ribosome, structural molecular activity, and ADP binding (Figure 5D, Supplementary Datasheet S15). “Structural component of the ribosome” was shared by all ecotypes and “RNA binding” was shared by all except ES12 (Figure 5D, Supplementary Datasheet S15).

The different ecotypes had 218 to 463 DEDMGs, of which 68% to 76% were ecotype-specific (Figure 5C). Some DEDMGs were shared between ecotypes, including four DEDMGs shared by all ecotypes: a SERPIN family protein serine protease inhibitor, a tubulin alpha-5 protein, a DNA-binding protein HEXBP-like, and a sieve element occlusion amino-terminus protein (Figure 5E).

**Figure 6:**
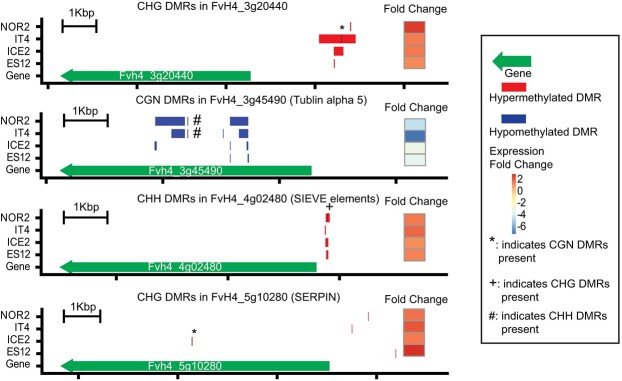
Functional analysis of DEDMGs in *Fragaria vesca* ecotypes propagated for three asexual generations at 18°C or 28°C. Graphs show expression and methylation status of four DEDMGs on *F. vesca* chromosome 4 (FvH4) that are shared by all four ecotypes (ES12, ICE2, NOR2, IT4). Red and blue bars represent hyper- and hypomethylated DMRs, respectively, in different methylation contexts (CHG, CGN, CHH, where N can be any base and H can be any base other than G). ^*^, +, and # indicate presence of CGN, CHG, and CHH DMRs, respectively, at the positions indicated by the black vertical lines.

For these four shared DEDMGs we plotted the location of DMRs relative to gene body and promoter regions and compared this with gene expression data. The expression change was going in the same direction in all four ecotypes. Three DEDMGs were hypermethylated and at the same time upregulated at 28°C, whereas one was hypomethylated and downregulated (Figure 6). Hypermethylated and upregulated DEDMGs were methylated in the CHG and CHH contexts in regulatory regions, whereas the hypomethylated and downregulated DEDMG was methylated in the CGN context in the gene body (Figure 6). Strikingly, the differential methylation patterns were very similarly positioned across ecotypes in all hypo- and hypermethylated DEDMGs, except for the SERPIN family protein FvH4_5g10280.

### Ecotypes show unique methylation and gene expression patterns in specific functions and pathways

After characterizing the shared DEDMGs, we examined ecotype-specific changes in genes related to the epigenetic machinery, flowering-time, temperature-regulation or gibberellin biogenesis and regulation in *A. thaliana*. We found 1318 associated DEDMGs with an absolute fold-change >1.5 (*p*_adj_ < 0.05) in three or fewer ecotypes (Supplementary Datasheet S14). Increasing the stringency to an absolute fold-change >2.0 reduced the number of DEDMGs to 678 (Supplementary Datasheet S14).

Methylation and expression of several *F. vesca* genes related to the epigenetic machinery in *A. thaliana* differed between plants propagated at 28°C versus 18°C (Supplementary Datasheet S14, Supplementary Figure S21). The ES12 and IT4 ecotypes shared upregulation of a gene encoding a homolog of an *A. thaliana* SWIB/MDM2 chromatin remodeling complex protein (FvH4_1g05690). ES12 and NOR2 shared upregulation of an *A. thaliana* chromatin reader Tudor-domain SAWADEE homolog (FvH4_6g40530) involved in RNA-directed DNA methylation in *A. thaliana* (Supplementary Figure S21). Other differentially regulated genes in individual ecotypes included a gene related to the *A. thaliana* structural maintenance of chromosomes (SMC) family (FvH4_7g27644 in ES12) that in *A. thaliana* participates in higher-order chromosome organization and dynamics, an *A. thaliana* XH/XS domain-containing protein homolog (FvH4_3g36253 in ICE2) involved in *A. thaliana* dsRNA binding in RdDM-related siRNA biogenesis, another *A. thaliana* SAWADEE protein homolog (FvH4_3g20410 in IT4), and an *A. thaliana* SETD group SET domain-containing protein gene and a PHD finger protein gene (FvH4_7g20720 and FvH4_4g12240 in NOR2) (Supplementary Figure S21).

Because temperature affected flowering time in NOR2 and ICE2, we examined changes in methylation and expression of genes homologous to flowering time genes in *Arabidopsis* (FlorID database) and other putative flowering-related genes (Supplementary Figure S22). The ICE2 and NOR2 ecotypes shared downregulation of an *A. thaliana ELONGATED HYPOCOTYL5 (HY5)* homolog (FvH4_4g21800) that is putatively involved in the epigenetic regulation of flowering [59], and an *A. thaliana* TCP transcription factor-homolog, a putative interactor of FLOWERING LOCUS T (FT) [60]. ES12 had downregulation of two *JOINTLESS-like/SVP/AGL22* homologs (FvH4_5g35400 and FvH4_5g35401), while ICE2 had upregulation of a *A. thaliana AGAMOUS-LIKE-24 (AGL24)* homolog (FvH4_3g03630). Both genes are involved in SOC1-dependent regulation of flowering in *A. thaliana* [61]. Furthermore, homologs of *A. thaliana* homeobox-containing protein genes linked to flowering were also identified; two in ES12 (FvH4_3g16431 and FvH4_5g27650) and two in ICE2 (FvH4_4g09960 and FvH4_7g23561). ES12 had an *A. thaliana* circadian clock-related B-box-containing protein homolog (FvH4_6g43580), a *VERNALIZATION* homolog (VRN1, FvH4_7g07610), and a *WRKY70-like* homolog (FvH4_7g26030) (Supplementary Datasheet S14).

Thirteen temperature-related DEDMGs were detected between plants propagated at 28°C versus 18°C, including genes encoding chaperones, protease activity, and MEDIATOR of RNA polymerase II subunit. Six DEDMGs were shared by pairs of ecotypes (ES12-NOR2 and ICE2-NOR2), while the remaining DEDMGs were found only in one ecotype (Supplementary Datasheet S14, Supplementary Figure S23), suggesting that these temperature-related DEDMGs do not represent a general temperature response.

Previous studies showed that GA activates stolon formation in *F. vesca,* and low activity of GA biosynthetic genes was associated with early flowering [62–64]. Therefore, we examined the expression and methylation patterns of gibberellin genes (Supplementary Figure S24). Interestingly, ICE2, IT4, and NOR2 shared a DEDMG encoding a CYP72A15 homolog (FvH4_3g17260) that reduces GA activity by catalyzing 13-hydrolyzation of gibberellic acid (GA) in *A. thaliana* [65]. The CYP72A15 homolog DEDMG was upregulated in all ecotypes affected (Supplementary Figure S24), thus suggesting reduced GA levels. In line with this, the same ecotypes tended to produce less stolons after the warm temperature treatment, although the difference was statistically significant only in one ecotype. Unexpectedly, the ICE2, IT4, and NOR2 ecotypes also flowered late after the warm temperature treatment indicating that other factors delayed flowering. We observed that NOR2 also had an additional differentially expressed and methylated CYP72A15 homolog (FvH4_7g17900). Other GA-related DEDMGs were unique to specific ecotypes: a GA receptor (FvH4_2g32670), a GA-regulated protein (FvH4_2g24370), and a GA 2-oxidase (FvH4_3g05530) in ICE2, a GA-regulated protein (FvH4_5g01680) in IT4, and a GA-STIMULATED ARABIDOPSIS 6 homolog (FvH4_5g14950) in NOR2 (Supplementary Datasheet S14, Supplementary Figure S24).

### REs may impact the expression of nearby genes

REs are well-known targets of DNA methylation, and RE methylation may affect the expression of nearby genes. We examined the methylation status of REs in the *F. vesca* genome, starting by comparing DMRs across temperature conditions. Most DMRs situated within REs were hypermethylated in plants propagated at 28°C versus 18°C, particularly in the CHG and CHH contexts (Supplementary Figure S25). We classified genes into four categories based on the distance between the gene and the closest RE: [1] no REs within 10 kbp, [2] an RE within 2 kbp, [3] an RE within 2 to 5 kbp, and [4] an RE within 5 to 10 kbp. Only REs within 2 kbp from genes had a significant correlation with transcription level (Games–Howell test, 0.05 > *p* > 0.001) (Supplementary Figure S26). The general effect of having an RE 2 kbp or less from a gene was associated with a significantly reduced transcript level of that gene (Supplementary Figure S26). Genes with REs close-by had a significantly greater change in transcript level than genes with no REs nearby (DEG at 28°C vs. 18°C, abs_log2FC, Games-Howell test, 0.001 ≥ *p* ≥ 0.005) (Supplementary Figure S25,S26, Supplementary Datasheet S16).

## Discussion

In this study, we examined how warmer temperatures are associated with DNA methylation during asexual reproduction in *F. vesca*. Methylomes and transcriptomes from four ecotypes with distinct phenologies were analyzed to identify DNA methylation changes and their association with gene expression. We also explored correlations between methylome signatures and phenological changes associated with exposing plants to different temperature conditions during asexual propagation. Our findings shed light on how perennial plants can adapt to a rapidly warming world, and how the methylome and transcriptome contribute to phenotypic variation.

### Temperature conditions induce increased phenotypic variation

The phenology of *F. vesca* plants was affected by the temperature conditions experienced during asexual reproduction. Exposure to warmer temperatures (28°C) during vegetative propagation delayed the time to flowering in the high-latitude ecotypes NOR2 and ICE2, and the effect increased from the first to the third asexual generation. An epigenetic memory of temperature conditions experienced during asexual reproduction has been observed previously in gymnosperm plants. Exposure to different temperatures during Norway spruce embryogenesis (a form of asexual reproduction) induces epitypes with lasting, reproducible, and predictable changes in adult tree traits, such as bud phenology and frost tolerance [35, 36]. Although an effect of temperature on flower induction of *F. vesca* is well established [66], our study is the first indication of an epigenetic memory-response caused by temperature conditions during asexual reproduction. The low-latitude ecotypes IT4 and ES12 did not show a significant impact on flowering. The shorter time to flowering and increased phenotypic variation observed in NOR2 and ICE2 indicate an epigenetic adaptation to shorter growth periods at high latitudes. The fact that the altered phenotype was detectable after three asexual generations indicates that effects on flowering time may accumulate over several generations at warmer temperature conditions.

The impact of temperature on stolon formation (in ES12 and IT4) and petiole length (in ES12, ICE2 and NOR2) was strongest in the first asexual generation but remained significant for three generations in IT4 (for stolon number) and NOR2 (for petiole length). Thus, there was a possible epigenetic impact of temperature on these adaptive features, but it was weaker than for flowering. Taken together, the different phenotypic responses to propagation temperature we observed between ecotypes suggest that ecotypes differ in plasticity. NOR2 was the most plastic ecotype, with a significant temperature memory affecting both flowering time and petiole length. We never observed significant flowering time difference and stolon number differences within the same ecotype, which points to antagonism between these reproductive modes and the potential trade-off of sexual reproduction (flowering time) and asexual reproduction (stolon) [67]. Low latitude ecotypes tend to have stolon differences that might hint that asexual reproduction dominates the fitness to environment, whereas high latitude ecotypes tend to be adapted to sexual reproduction. Many theories have tried to explain the genetic and ecological fitness associated with sexual reproduction versus asexual propagation, but the evolution of reproductive systems, their true adaptive value and costs associated with them, remains an unresolved puzzle in biology [68].

### Plants exposed to warmer temperatures undergo massive changes in DNA methylation

We used whole genome bisulfite sequencing to probe changes in the methylomes of four *F. vesca* ecotypes in response to warmer temperatures (28°C vs. 18°C). All ecotypes exhibited individual hyper- and hypomethylated peaks at 28°C, indicating that there were numerous global and specific methylation differences between ecotype methylomes. The NOR2 ecotype, being adapted to the coldest climate in this study, had the largest methylation change and seemed to have the most plastic methylome of the four ecotypes we studied. NOR2 showed significant methylation changes in all genic features (gene body, promoter, 3′ region) in all methylation contexts. This mirrors the higher phenotypic plasticity we observed in NOR2. In line with this finding, Li and co-workers demonstrated that high temperature sensitive accession of Rice (*Oryza sativa*) showed significantly increased DNA methylation levels compared to temperature resistant accessions [69]. In contrast, in similar experiments in Cotton (*Gossypium hirsutum*) higher DNA methylation in the CHH context was observed in heat-tolerant accessions compared to heat-susceptible accession upon heat treatment [70].

RE and pseudogenes showed significant methylation changes in all ecotypes for all methylation contexts upon temperature treatment, in contrast to the more subtle and selective methylation changes observed in other genic features. Correspondingly, in cucumber (*Cucumis sativus*) low temperature treatment resulted in DNA demethylation, especially in the CHH context [71] and a similar response has been described in mulberry (*Morus notabilis*) and Arabidopsis following pathogen infection [72], and *F. vesca* following multiple stresses [106], suggesting a common methylation response of transposons to both biotic and abiotic impacts.

CHG and CHH DMRs were mostly located in intergenic regions and in the 5′ direction of the transcription start site (TSS), whereas CGN DMRs mostly were located in promoter and gene body regions. In plants, the gene body is usually enriched with CGN methylation, whereas CGN methylation is depleted at the transcriptional start and termination sites. Similar to our findings, genes with high CGN gene body methylation are usually highly expressed and conserved [20, 73–77].

The CHG methylation context had most DMRs and DMGs, whereas CGN was both hypomethylated and hypermethylated in response to warmer temperatures. Most temperature-associated CHG and CHH DMRs were hypermethylated in all ecotypes. This indicates that CHG and CHH methylation and maintenance pathways are involved in *F. vesca* responses to temperature (CMT3 for CHG methylation and CMT2 and DRM1/DRM2 through RdDM for CHH methylation) [78]. We speculate that the temperature-associated hypermethylation in the CHG and CHH contexts could be enhanced by “self-reinforcing” chromatin interaction mediated interplay of histone methyltransferases (KYP, SUVH5/6) and CMT2/3 [24–27].

### Shared DMR peaks between ecotypes identify “target of XLP2 genes” as possible epigenetic targets

We observed several regions with a high density of DMRs, denoted DMR peaks. These mostly differed between ecotypes, but shared DMR peaks existed on *F. vesca* chromosomes Fvb2, Fvb3, Fvb5, and Fvb6, as previously reported [106]. Only eight peaks were shared by all ecotypes, and all these were in the CHG context. We found five TPX2 genes inside three DMR peaks, with three having homologs in Arabidopsis. The TPX2 homologues in *F. vesca* are involved in spindle formation in hormonal regulation of hypocotyl cell elongation, light signaling, vascular development, and abiotic stress tolerance [79]. Whether increased methylation of TPX2 genes is a direct or indirect effect of temperature-associated impacts on cell division remains to be determined, since TXP2 expression was changed in only three out of four ecotypes. It is, however, interesting that five (out of 21) members of the same protein family were situated within DMR peaks that make up less than 0.2% of the *F. vesca* genome (0.4 Mb/240 Mb). Their high methylation level suggests that these peak regions, displaying normal CG levels, may contain common recognition features that are targeted by the epigenetic machinery following exposure to warmer temperatures. All DMR peaks were enriched in the CHG context, suggesting that these peak regions can be used to uncover the mechanistic pathways by which environmental stressors target CHG DNA methylation.

### Methylation responses to warmer temperatures are accompanied by transcriptional changes

Independent of the ecotype, we identified 2500 to 4000 temperature-associated DMGs that were mostly ecotype-specific and connected to increased CHG methylation. Both DMGs and phenotypic responses were thus ecotype-specific. NOR2 had the highest number of DMGs and the strongest associated methylation response to warmer temperatures.

Transcriptomic changes associated with warmer temperature was observed in about 3500 to 5000 DEGs in the different ecotypes, with 8% to 18% of these being DEDMGs. These DEDMGs were statistically overrepresented compared to random gene samples, suggesting that the associated DNA methylation impacted the expression of at least a subset of these DEGs. Translation-related GO terms were enriched in DEDMGs in all four ecotypes, indicating that DNA methylation impacted the regulation of the translational machinery. Higher temperatures relax RNA secondary structure, resulting in more efficient translation initiation through the RNA thermoswitch [80, 81]. We observed a downregulation of genes directly linked to ribosomal subunits which may suggest the existence of a compensatory mechanism to maintain equilibrium within the translation process, counteracting the potential promotion of translation caused by warmer temperature.

Only four DEDMGs occurred in all four ecotypes with the same DNA methylation context and the same genomic location. The co-localization of DNA methylation within these DEDMGs suggests a mechanism that recognizes specific genic regions and leads to DNA methylation in response to high temperature. Previous studies in lotus (*Nelumbo nucifera*) also reported a similar correlation of methylome and gene expression changes between ecotypes with up to 20% of 1500 to 2500 genes showing correlation [82].

### DEDMGs and observed plant phenotypes

Our DNA methylation and gene expression analyses revealed that each ecotype was associated with unique sets of DMRs, DMGs, and DEDMGs when exposed to warmer temperatures.

Several flowering-associated DEDMGs were detected in NOR2 and ICE2. For example, ICE2 DEDMGs included an *FT* homologous gene and homologs of *A. thaliana* AGAMOUS and AGL24, VRN1, and HY5. NOR2 DEDMGs included homologs of *A. thaliana PHYTOCHROME AND FLOWERING TIME 1* and *HY5*. The *HY5* homologs were downregulated in ICE2 and NOR2. In *A. thaliana,* HY5 together with HISTONE DEACETYLASE 9, directly binds to the promoter of PHYTOCHROME INTERACTING FACTOR 4 and CONSTANS LIKE 5 that promote flowering in short days [59, 83]. Downregulation of HY5 could, thus, explain the delayed flowering in the ICE2 and NOR2 ecotypes in response to warmer temperatures. Although temperature did not affect flowering time in ES12 and IT4, two MADS-box JOINTLESS-like/SVP/AGL22 transcription factors homologs and an *A. thaliana* SAWADEE homolog involved in flowering were identified as DEDMGs in ES12 and IT4, respectively [84, 85]. As these two ecotypes hardly flowered at all in our study, it is possible that these DEDMGs play a more significant role in flowering under natural conditions, where IT4 and ES12 perhaps would flower easily.

DEDMGs in ICE2 and NOR2 may be involved in the observed epigenetic memory effect on flowering time, although more detailed gene expression analyses during flower-induction are required to clarify their potential impact. FvFT1, a known repressor of flowering in *F. vesca* [63, 86, 87] was downregulated >10-fold at warmer temperatures and correlated with delayed flowering in ICE2. However, AGL24 and VRN1, which promote flowering in Arabidopsis [88, 89], were downregulated in ICE2 and showed a positive correlation between gene expression and the observed flowering phenotype.

### Temperature-associated methylation of the epigenetic machinery

We found 21 DEDMGs that are involved in the DNA methylation machinery, either directly or indirectly through effects on other chromatin modifications. Only four of these were shared by at least two ecotypes, suggesting that DNA methylation associated with warmer temperatures ultimately could lead to further chromatin changes in an ecotype-specific manner. Examples of DEDMGs with roles in the DNA methylation machinery were discussed above, including the aforementioned SAWADEE homolog involved in RNA-directed DNA methylation, a small RNA-degrading nuclease [84, 90], and Jmjc histone demethylases, such as the JMJ20, that may reinforce histone H3K9me2 marks and enhance CMT3/2 CHG/CHH methylation [91, 92].

## Concluding remarks

We have demonstrated a clear memory effect of warmer temperatures at the phenotypic level in *F. vesca*. This was accompanied by changes in DNA methylation and transcriptional changes. Unlike the common garden phenotypic analysis, sampled plant material for methylation and expression studies were exposed to continuous temperatures and due to this experimental setup a memory effect cannot be concluded for these. The temperature memory effect is ecotype (genotype)-specific and some ecotypes were more plastic than others. To this end, the NOR2 ecotype displayed a significant temperature memory affecting both flowering time and petiole length that could be correlated to specific DNA methylation and expression changes.

## Materials and methods

### Plant material and experimental conditions

*F. vesca* ecotypes (genotypes) “ES12” (43.5339°N, 6.5271°W, 138 m, Spain), “ICE2” (63.9988°N, 19.9604°W, 99 m, Iceland), “IT4” (46.2398°N, 11.2360°E, 949 m, Italy), and “NOR2” (69.9395°N, 23.0964°E, 23 m, Norway) were propagated as clones from stolons (Supplementary Figure S1). Clonal descendants from one individual plant per ecotype were grown in a growth chamber equipped with Valoya AP67 LED lamps (200 μmol∙m^−2^∙s^−1^ photosynthetically active radiation (PAR) at 40–45% humidity). A long day (LD) length of 16 h/8 h (light/dark) was used for plant vegetative growth and a short day (SD) length of 12 h/12 h was used to induce flowering. Plants were grown in 400 ml pots and fertilized regularly with a fertilizer solution (N-P-K: 17-4-25, Kekkilä, Finland). Up to three asexual generations were generated at normal temperatures (18°C) and at warmer temperatures (28°C) (Figure 1B). For the first asexual generation (AS1), stolons from mother plants of the four ecotypes were rooted at both temperatures. Subsequent asexual generations (AS2 and AS3) were generated with 3-week intervals using stolons from previous asexual generations. Two weeks after the initiation of AS1 and AS3, 6 hours after lights were turned on, 20 mg of young, unfolded leaf tissues was collected from 10 plants per temperature condition per ecotype for DNA/RNA isolation. From these 10 plants we randomly selected three independent biological replicates (individual plants) for sequencing library construction. Three weeks after leaf sampling, plants were moved to SD conditions at 18°C for 5 weeks (AS1) or 6 weeks (AS3) to induce flowering. After SD treatment, plants were moved to a common environment (16/8 h LD, 18°C) in the greenhouse for further phenotypic observations. For phenotypic observation, ten plants were recorded for each ecotype and each treatment (18°C/28°C). Petiole length of the youngest fully developed leaf was recorded 3–5 weeks after the start of the common garden environment. Flowering time was recorded as days to the first open flower after the end of SD treatment, for a duration of two to nine weeks. The number of stolons produced was recorded every two weeks for 14 weeks starting at week two. AS2 plants were not sampled for molecular analyses. Statistical comparisons of phenotypic parameters between ecotypes and temperatures were done using the Wilcoxon test in R.

### DNA and RNA isolation

Simultaneous extraction of both genomic DNA and total RNA from leaves was performed as described previously [93].

### Bisulfite and RNA-sequencing

Bisulfite library preparation and sequencing were done by BGI using the HiSeq platform. BGI also constructed transcriptome libraries and performed RNA-seq using the DNB-seq platform. Three independent replicates of bisulfite reads were mapped and analyzed using the default settings in the methylpy pipeline [94]. Trimmed reads were aligned to the *F. vesca* reference genome (v4.0) [44] using the bowtie2 read-aligner that is implemented in methylpy [95]. PCR duplicate reads were removed in a de-duplication step using MarkDuplicates (Picard suite v.2.18.1, https://github.com/broadinstitute/picard). Principal component analysis (PCA) and other analyses of global DNA methylation patterns and methylation of different ecotypes and temperature epitypes were done using methylpy and R with a window size of 50 kb [94]. Global methylation circos plots were made using shinyCircos with methylation data from the global methylation analysis [96]. Plots of gene densities and methylation levels were made using the R package Rideogram [97]. Statistical comparisons of methylation of different genomic features were done using the Wilcoxon test in R. DMRs of different methylation contexts were identified using the DMRfind function of methylpy. First, differentially methylated sites (DMSs) were called using a root mean square test with a false discovery rate of 0.003, a minimum read coverage of 30, and 1000 permutations [94], using chloroplast sequences as an unmethylated control. All DMSs located within 250 bps were merged and considered to constitute a single DMR. The distribution of methylation sites across genes and REs were plotted using the R package methimpute [98]. Gene body regions and REs were divided into 20 bins to calculate the methylation degree, whereas flanking regions (±2 kb) were sectioned into 20 pieces of 100 bp each. DMRs were annotated to genomic features using the R package ChIPseeker [99]. DMR circos plots were made using shinyCircos [96]. Venn plots were generated using an online tool (https://bioinformatics.psb.ugent.be/webtools/Venn/).GO term enrichment analysis was done using the R package clusterprofiler [100] and GO term annotation (v4.0.a2) [45] was acquired from the genome database ROSACEAE (GDR, https://www.rosaceae.org/) [101]. Any positional effects of REs were analyzed using the Games-Howell test from the R package rstatix. Heatmaps were made using the R package pheatmap. The motif identification for DMRs and adjacent 200 bp region was done by using HOMER2 with default setting [53].

Three independent replicates of RNA-seq reads were analyzed using the default settings in CLC Genomics Workbench (Qiagen Ltd). The reads were mapped to the *F. vesca* reference genome (v4.0) [44] (Supplementary Datasheet S14). PCA of individual replicates was done using prcomp() in R using the transcript per million (TPM) value of each gene. Volcano plots were made using the ggplot2 package in R, based on log_2_FoldChange-values and log_10_ p-values from the CLC output. CLC uses trimmed mean of M values (TMM) for library size normalizations [102]. A generalized linear model was used to call differentially expressed genes (DEGs) and the Wald test was used to determine if DEGs differed statistically [103]. For statistical testing of overlapping DMGs and DEGs an online tool running Fisher’s exact test was used (http://nemates.org/MA/progs/overlap_stats.html). REs (DATASHEET1) were predicted using REPEATMODELER2 with default settings [104]. Pseudogenes (DATASHEET2) were predicted using pseudopipe [105]. Linear regression analysis to explore relationships between DNA methylation and genomic features was done in R (model: methylation~gene density + RE density + pseudogene density).

## Acknowledgements

We would like to thank Dr. Simeon Rossmann from NIBIO for help on R and Linux scripting. We also would like to thank Katriina Palm and Javier Andrés, both from the University of Helsinki, for taking care of our experimental plants and providing the flowering gene list, respectively. This work was supported by Norges Forskingsråd through a Toppforsk project 249958 “Beyond the genome: epigenetics of defense priming and climatic adaptation in plants”.

## Author contributions

C.G.F., P.E.G., and Y.P.Z. designed the analysis. C.G.F. designed the research. Y.P.Z. and G.X.F. performed experiments. Y.P.Z., G.X.F., T.T., T.Ts., I.Y., T.H., P.E.G., and C.G.F. analyzed the data. Y.P.Z., T.Ts., T.H., P.E.G., and C.G.F. discussed the data. Y.P.Z., P.E.G., and C.G.F. wrote the article. Y.P.Z., P.K., T.H., P.E.G., and C.G.F. revise the article. All authors approved the article.

## Data availability

All sequences generated in this study have been deposited in the National Center for Biotechnology Information Sequence Read Archive (https://www.ncbi.nlm.nih.gov/sra) under project number PRJNA879428. Supporting data and DATASHEETs are available at GitHub (github.com/sherlock0088/FvAsex).

## Conflict of interest statement

None declared.

## Supplementary Data

Supplementary data is available at Horticulture Research online.

## Supplementary Material

Web_Material_uhad156
