# Supplementary material for: Warmer temperature during asexual reproduction induce methylome, transcriptomic, and lasting phenotypic changes in Fragaria vesca ecotypes
Source: Hortic Res. 2023 Jul 31;10(9):uhad156. doi: 10.1093/hr/uhad156 (PMC10500154; doi:10.1093/hr/uhad156)
Supplement: Web_Material_uhad156 [file web_material_uhad156.zip › Supplementary Figure 16.pdf]

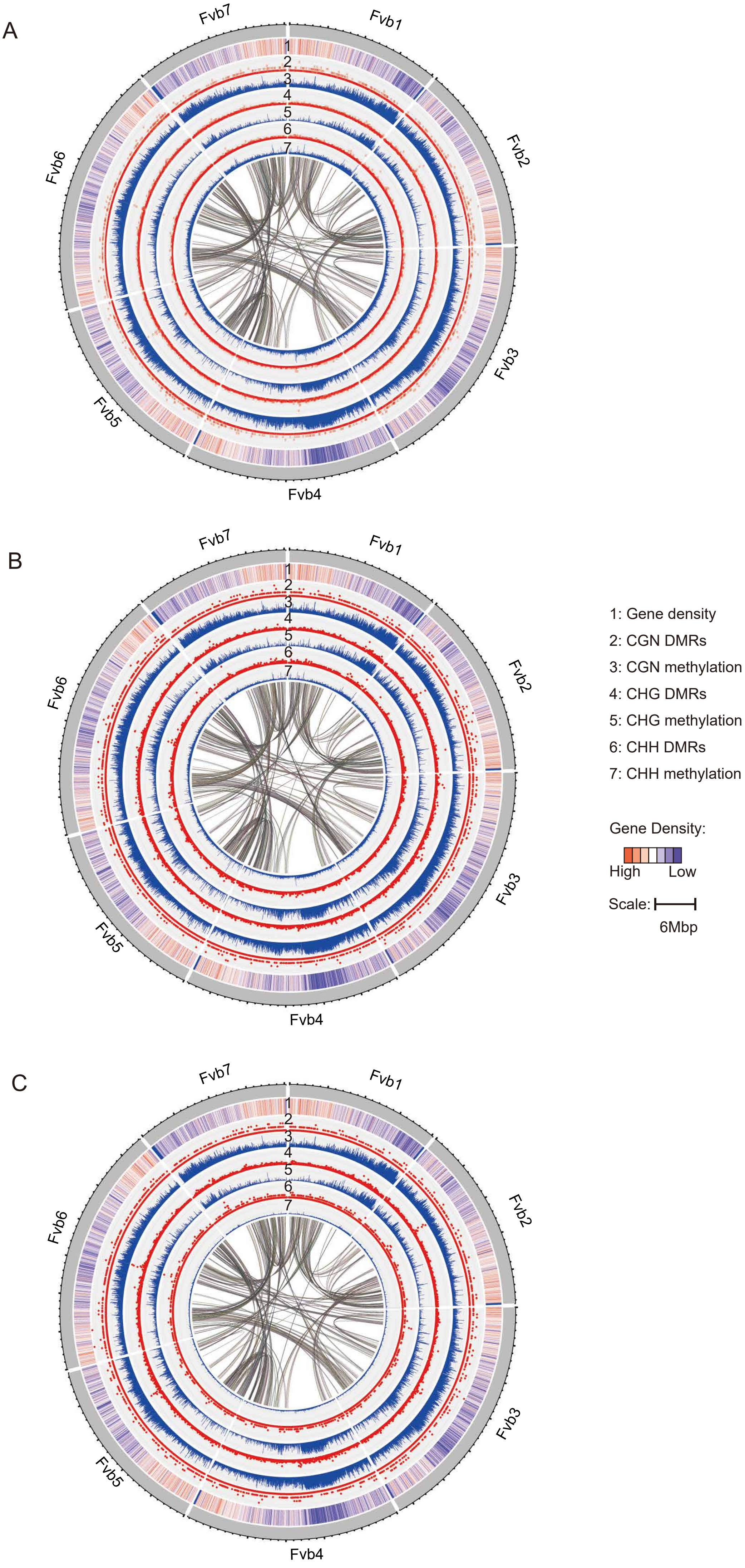

A
B
C
Fvb1
Fvb2
Fvb3
Fvb4
Fvb5
Fvb6
Fvb7
1: Gene density
2: CGN DMRs
3: CGN methylation
4: CHG DMRs
5: CHG methylation
6: CHH DMRs
7: CHH methylation
Gene Density:
High
Low
Scale:
6Mbp
