# Supplementary material for: Warmer temperature during asexual reproduction induce methylome, transcriptomic, and lasting phenotypic changes in Fragaria vesca ecotypes
Source: Hortic Res. 2023 Jul 31;10(9):uhad156. doi: 10.1093/hr/uhad156 (PMC10500154; doi:10.1093/hr/uhad156)
Supplement: Web_Material_uhad156 [file web_material_uhad156.zip › Supplementary Figure 21.pdf]

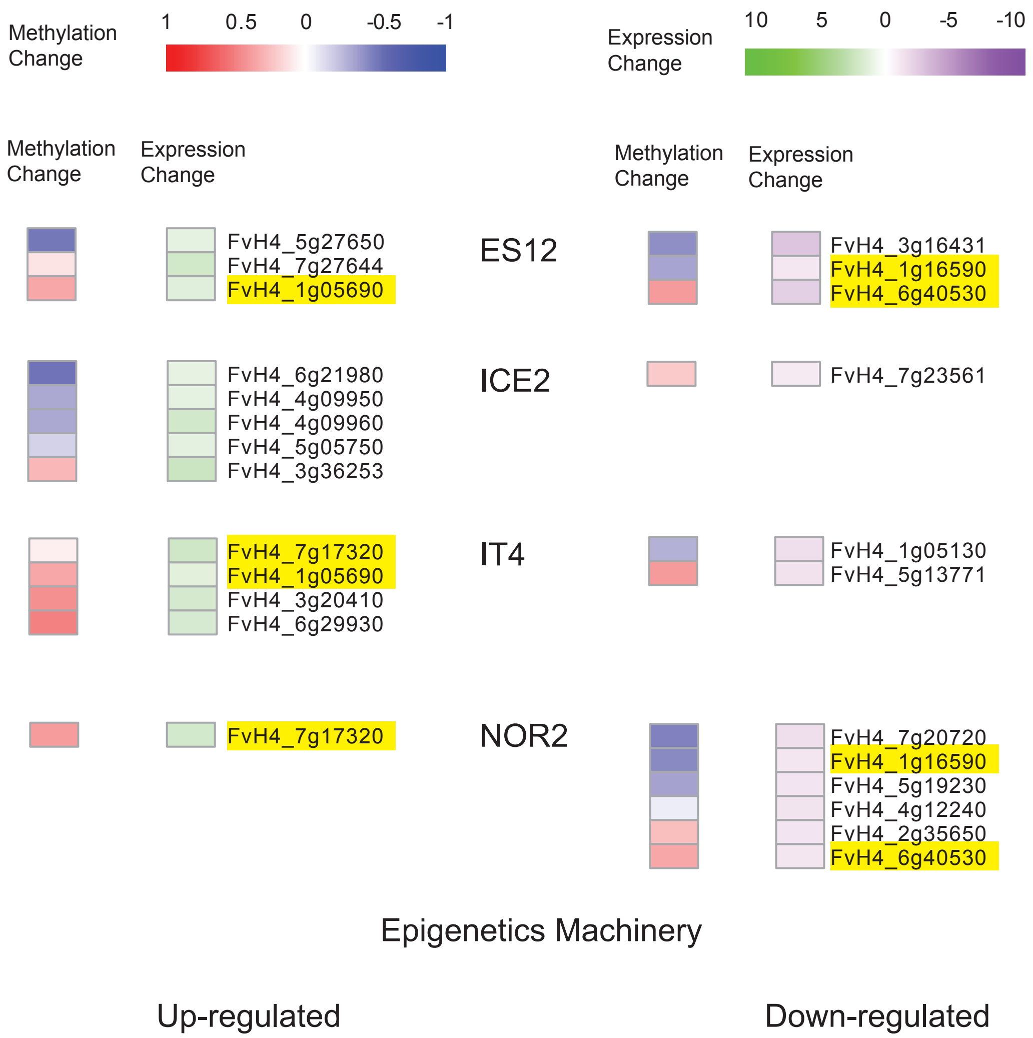

Methylation Change
1
0.5
0
-0.5
-1
Expression Change
10
5
0
-5
-10
Methylation Change
Expression Change
Methylation Change
Expression Change
FvH4_5g27650
FvH4_7g27644
FvH4_1g05690
ES12
FvH4_3g16431
FvH4_1g16590
FvH4_6g40530
FvH4_6g21980
FvH4_4g09950
FvH4_4g09960
FvH4_5g05750
FvH4_3g36253
ICE2
FvH4_7g23561
FvH4_7g17320
FvH4_1g05690
FvH4_3g20410
FvH4_6g29930
IT4
FvH4_1g05130
FvH4_5g13771
FvH4_7g17320
NOR2
FvH4_7g20720
FvH4_1g16590
FvH4_5g19230
FvH4_4g12240
FvH4_2g35650
FvH4_6g40530
Epigenetics Machinery
Up-regulated
Down-regulated
