# Supplementary material for: Warmer temperature during asexual reproduction induce methylome, transcriptomic, and lasting phenotypic changes in Fragaria vesca ecotypes
Source: Hortic Res. 2023 Jul 31;10(9):uhad156. doi: 10.1093/hr/uhad156 (PMC10500154; doi:10.1093/hr/uhad156)
Supplement: Web_Material_uhad156 [file web_material_uhad156.zip › Supplementary Figure 22.pdf]

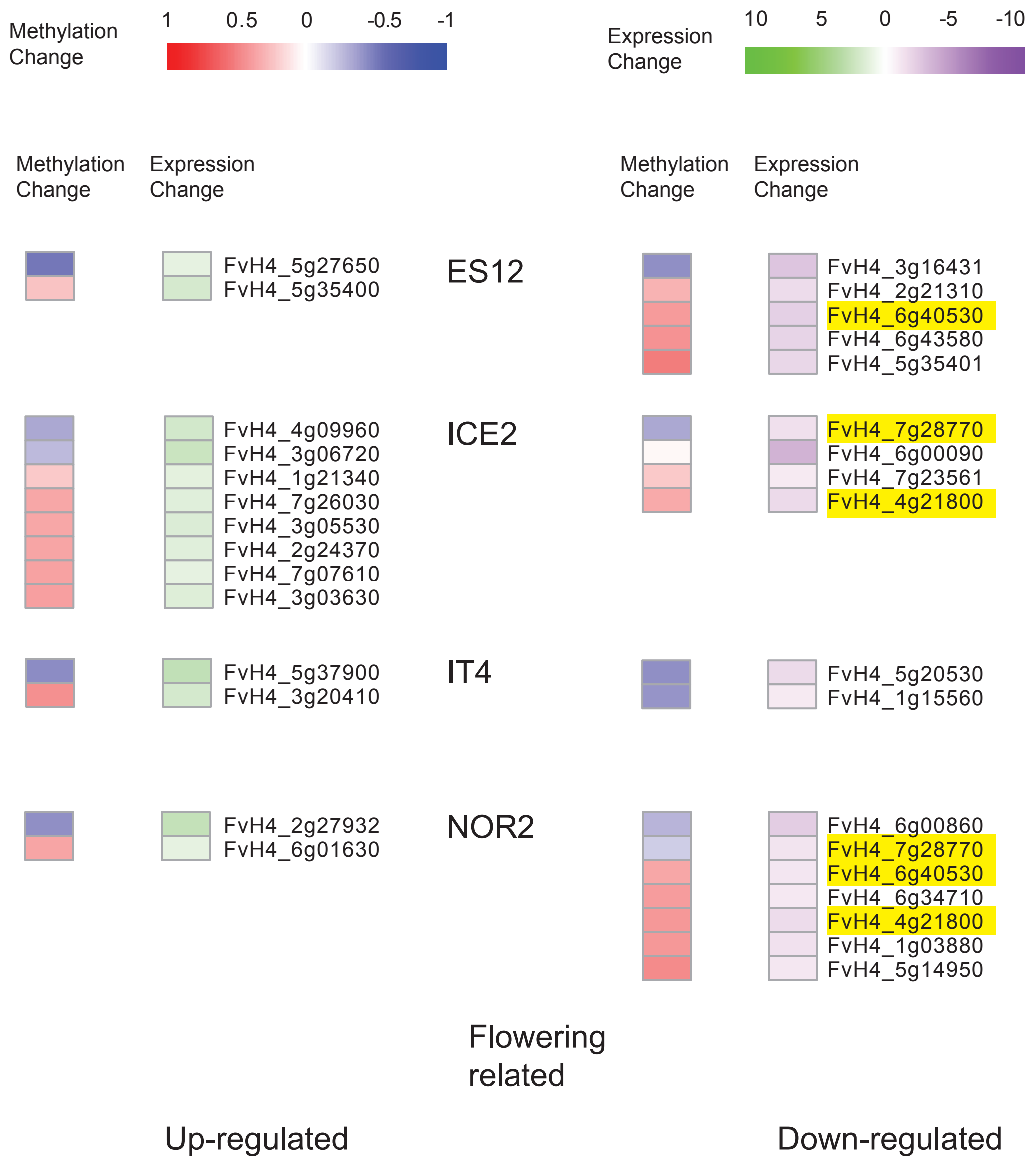
Methylation Change
1
0.5
0
-0.5
-1
Expression Change
10
5
0
-5
-10
Methylation Change
Expression Change
Methylation Change
Expression Change
FvH4_5g27650
FvH4_5g35400
ES12
FvH4_3g16431
FvH4_2g21310
FvH4_6g40530
FvH4_6g43580
FvH4_5g35401
FvH4_4g09960
FvH4_3g06720
FvH4_1g21340
FvH4_7g26030
FvH4_3g05530
FvH4_2g24370
FvH4_7g07610
FvH4_3g03630
ICE2
FvH4_7g28770
FvH4_6g00090
FvH4_7g23561
FvH4_4g21800
FvH4_5g37900
FvH4_3g20410
IT4
FvH4_5g20530
FvH4_1g15560
FvH4_2g27932
FvH4_6g01630
NOR2
FvH4_6g00860
FvH4_7g28770
FvH4_6g40530
FvH4_6g34710
FvH4_4g21800
FvH4_1g03880
FvH4_5g14950
Flowering related
Up-regulated
Down-regulated
