# Supplementary material for: Warmer temperature during asexual reproduction induce methylome, transcriptomic, and lasting phenotypic changes in Fragaria vesca ecotypes
Source: Hortic Res. 2023 Jul 31;10(9):uhad156. doi: 10.1093/hr/uhad156 (PMC10500154; doi:10.1093/hr/uhad156)
Supplement: Web_Material_uhad156 [file web_material_uhad156.zip › Supplementary Figure 23.pdf]

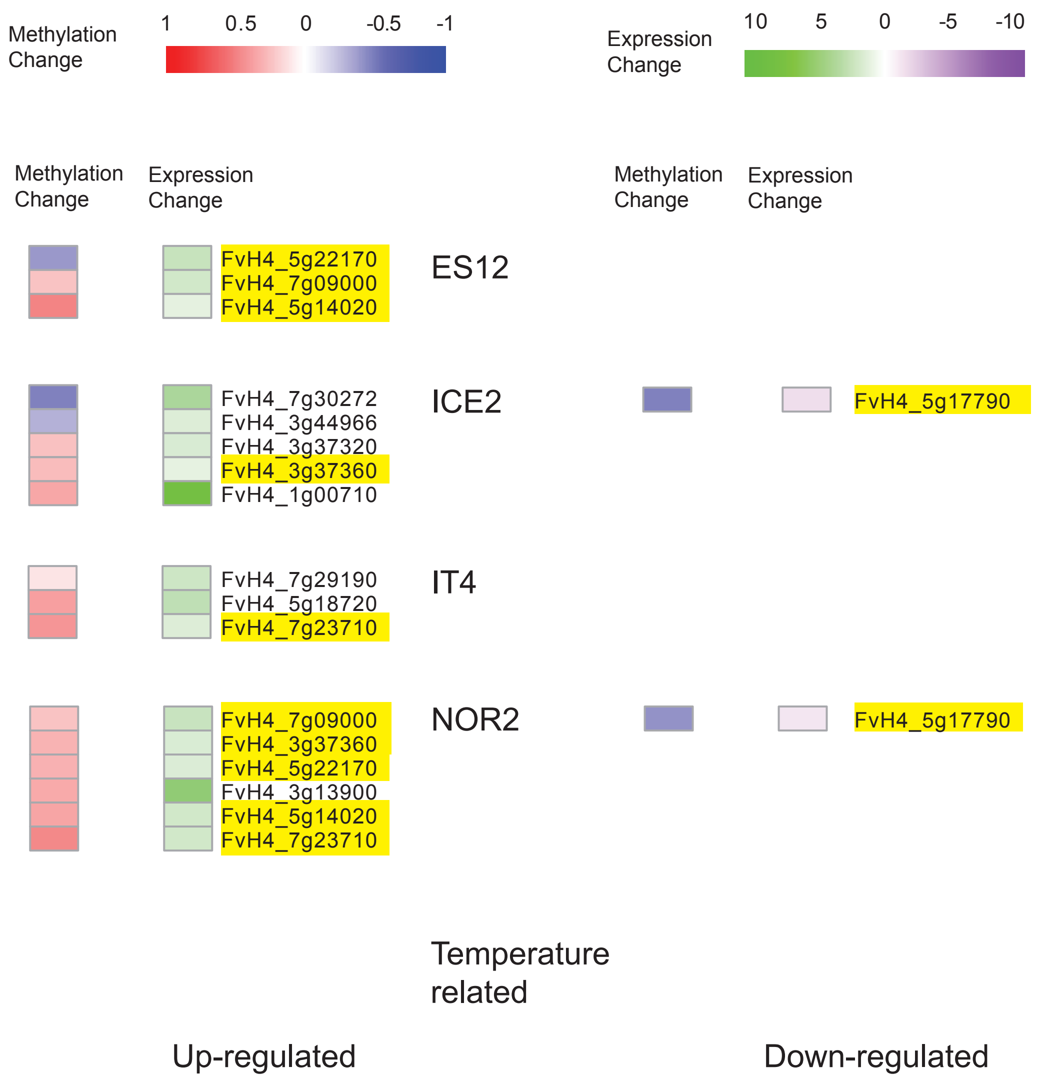
Methylation Change
1
0.5
0
-0.5
-1
Expression Change
10
5
0
-5
-10
Methylation Change
Expression Change
Methylation Change
Expression Change
FvH4_5g22170
FvH4_7g09000
FvH4_5g14020
ES12
FvH4_7g30272
FvH4_3g44966
FvH4_3g37320
FvH4_3g37360
FvH4_1g00710
ICE2
FvH4_5g17790
FvH4_7g29190
FvH4_5g18720
FvH4_7g23710
IT4
FvH4_7g09000
FvH4_3g37360
FvH4_5g22170
FvH4_3g13900
FvH4_5g14020
FvH4_7g23710
NOR2
FvH4_5g17790
Temperature related
Up-regulated
Down-regulated
