# Supplementary material for: Warmer temperature during asexual reproduction induce methylome, transcriptomic, and lasting phenotypic changes in Fragaria vesca ecotypes
Source: Hortic Res. 2023 Jul 31;10(9):uhad156. doi: 10.1093/hr/uhad156 (PMC10500154; doi:10.1093/hr/uhad156)
Supplement: Web_Material_uhad156 [file web_material_uhad156.zip › Supplementary Figure 24.pdf]

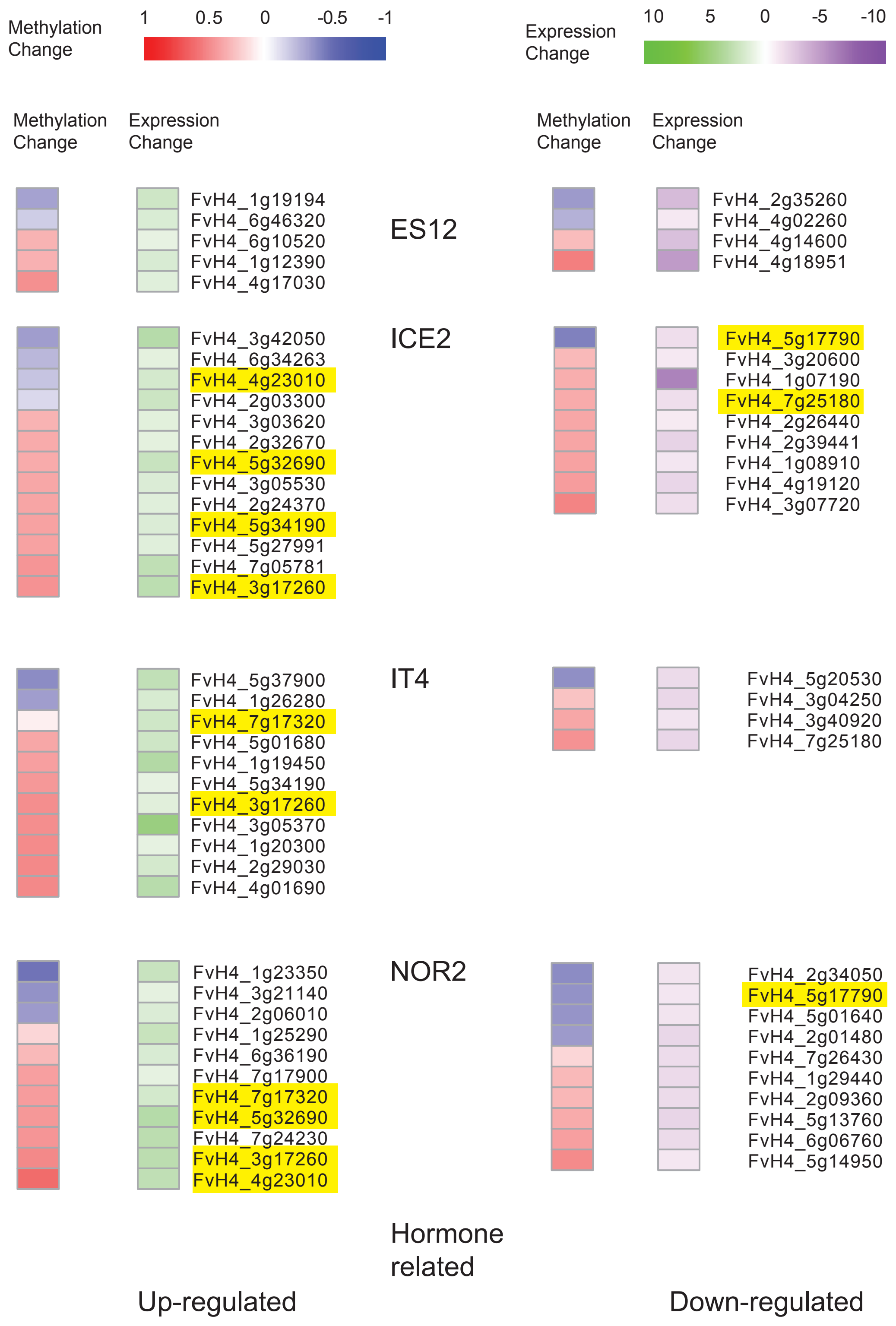
Methylation Change
1
0.5
0
-0.5
-1
Expression Change
10
5
0
-5
-10
Methylation Change
Expression Change
Methylation Change
Expression Change
ES12
FvH4_1g19194
FvH4_6g46320
FvH4_6g10520
FvH4_1g12390
FvH4_4g17030
FvH4_2g35260
FvH4_4g02260
FvH4_4g14600
FvH4_4g18951
ICE2
FvH4_3g42050
FvH4_6g34263
FvH4_4g23010
FvH4_2g03300
FvH4_3g03620
FvH4_2g32670
FvH4_5g32690
FvH4_3g05530
FvH4_2g24370
FvH4_5g34190
FvH4_5g27991
FvH4_7g05781
FvH4_3g17260
FvH4_5g17790
FvH4_3g20600
FvH4_1g07190
FvH4_7g25180
FvH4_2g26440
FvH4_2g39441
FvH4_1g08910
FvH4_4g19120
FvH4_3g07720
IT4
FvH4_5g37900
FvH4_1g26280
FvH4_7g17320
FvH4_5g01680
FvH4_1g19450
FvH4_5g34190
FvH4_3g17260
FvH4_3g05370
FvH4_1g20300
FvH4_2g29030
FvH4_4g01690
FvH4_5g20530
FvH4_3g04250
FvH4_3g40920
FvH4_7g25180
NOR2
FvH4_1g23350
FvH4_3g21140
FvH4_2g06010
FvH4_1g25290
FvH4_6g36190
FvH4_7g17900
FvH4_7g17320
FvH4_5g32690
FvH4_7g24230
FvH4_3g17260
FvH4_4g23010
FvH4_2g34050
FvH4_5g17790
FvH4_5g01640
FvH4_2g01480
FvH4_7g26430
FvH4_1g29440
FvH4_2g09360
FvH4_5g13760
FvH4_6g06760
FvH4_5g14950
Hormone related
Up-regulated
Down-regulated
