# Supplementary figures and images for: Warmer temperature during asexual reproduction induce methylome, transcriptomic, and lasting phenotypic changes in Fragaria vesca ecotypes
Source: Hortic Res. 2023 Jul 31;10(9):uhad156. doi: 10.1093/hr/uhad156 (PMC10500154; doi:10.1093/hr/uhad156)

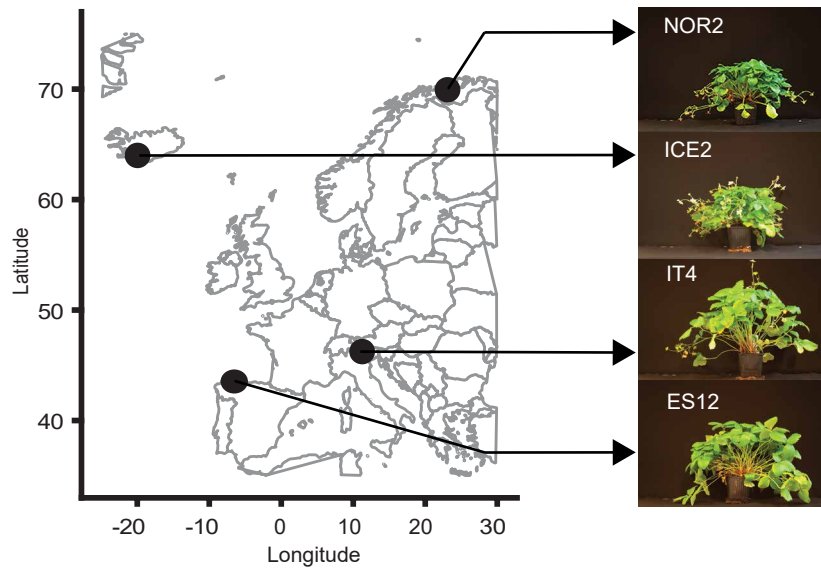

Supplement: Web_Material_uhad156 [file web_material_uhad156.zip › Supplementary Figure 1.pdf]

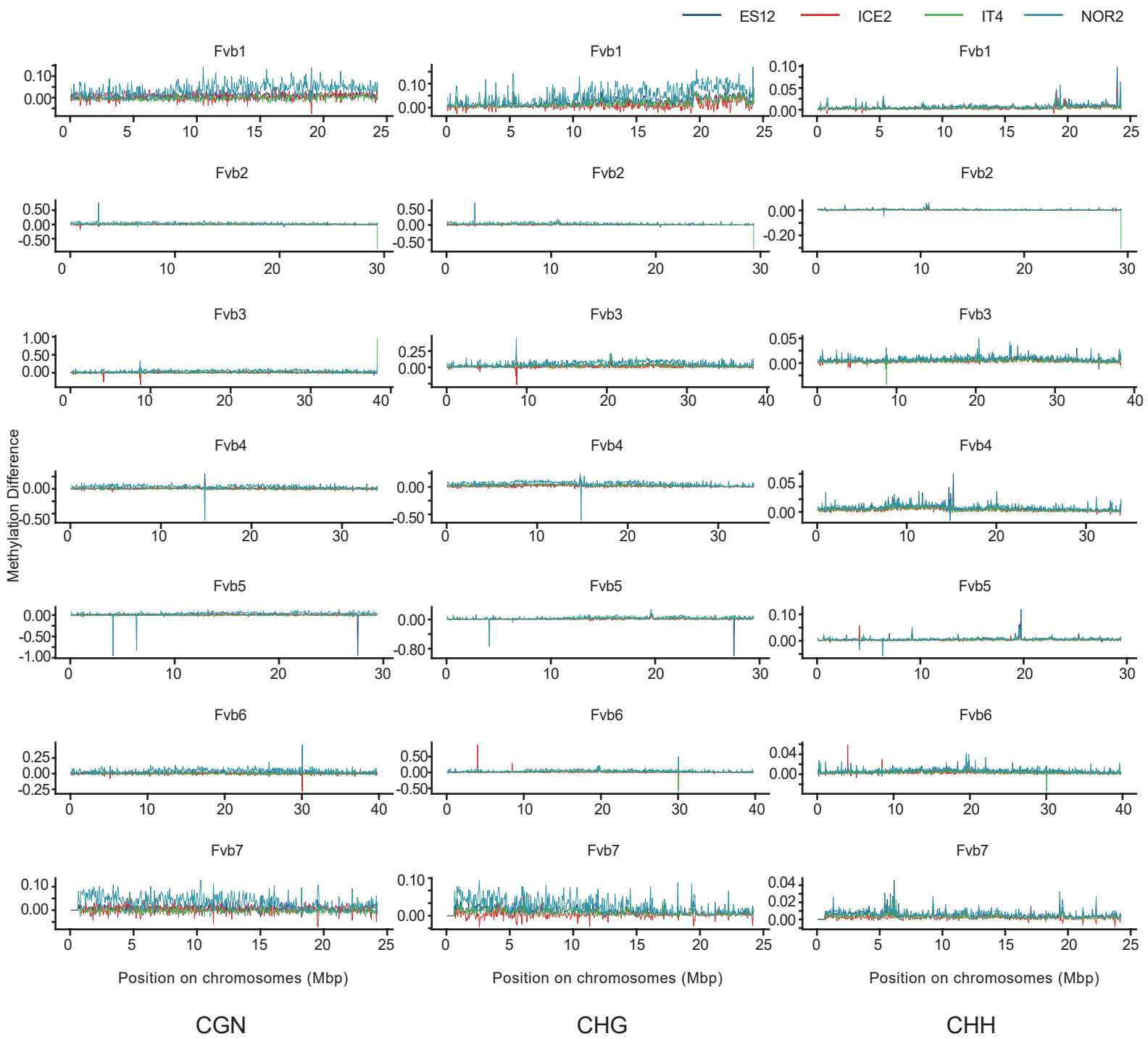

Supplement: Web_Material_uhad156 [file web_material_uhad156.zip › Supplementary Figure 10.pdf]

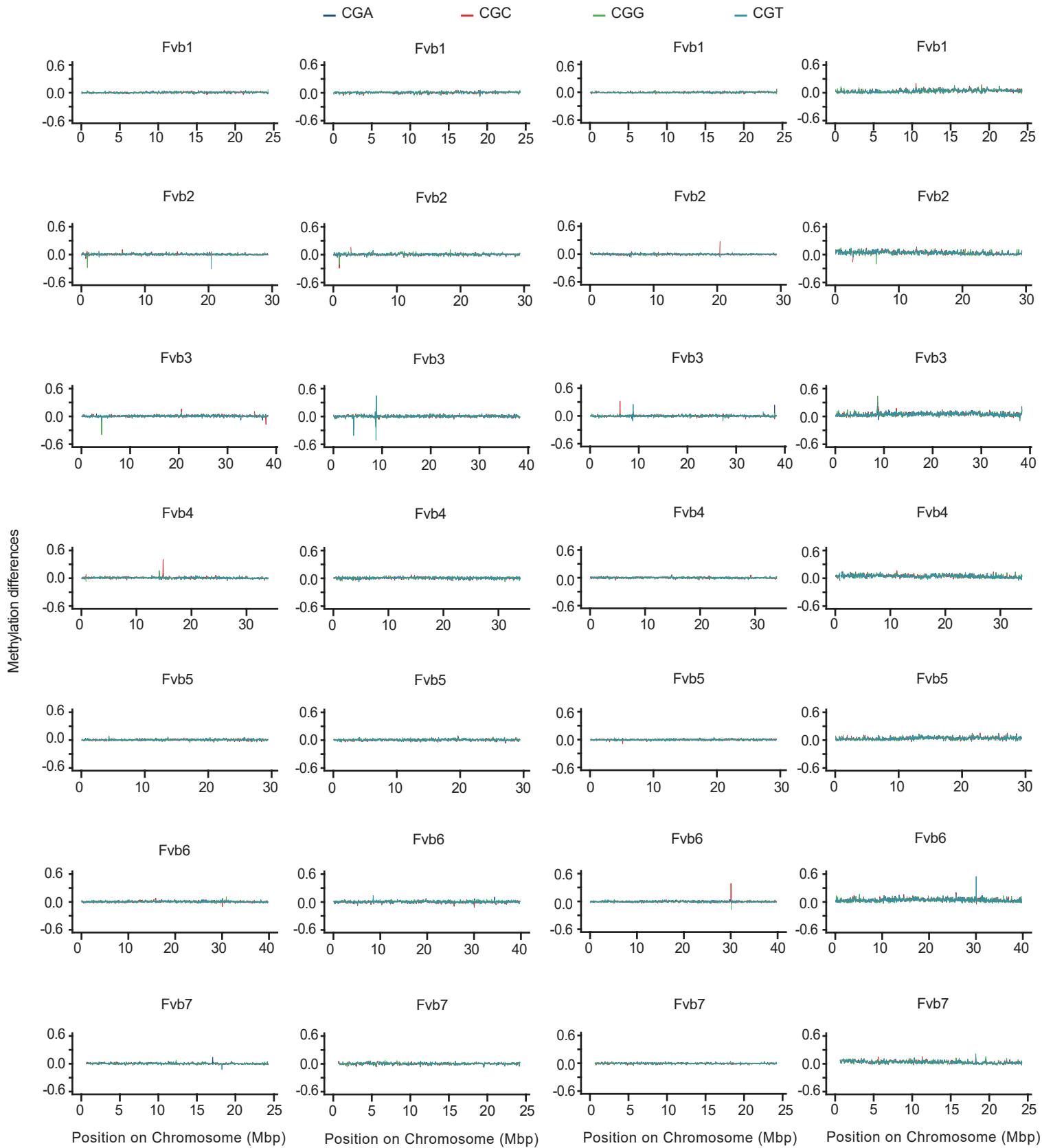

ES12

ICE2

IT4

NOR2

Supplement: Web_Material_uhad156 [file web_material_uhad156.zip › Supplementary Figure 11.pdf]

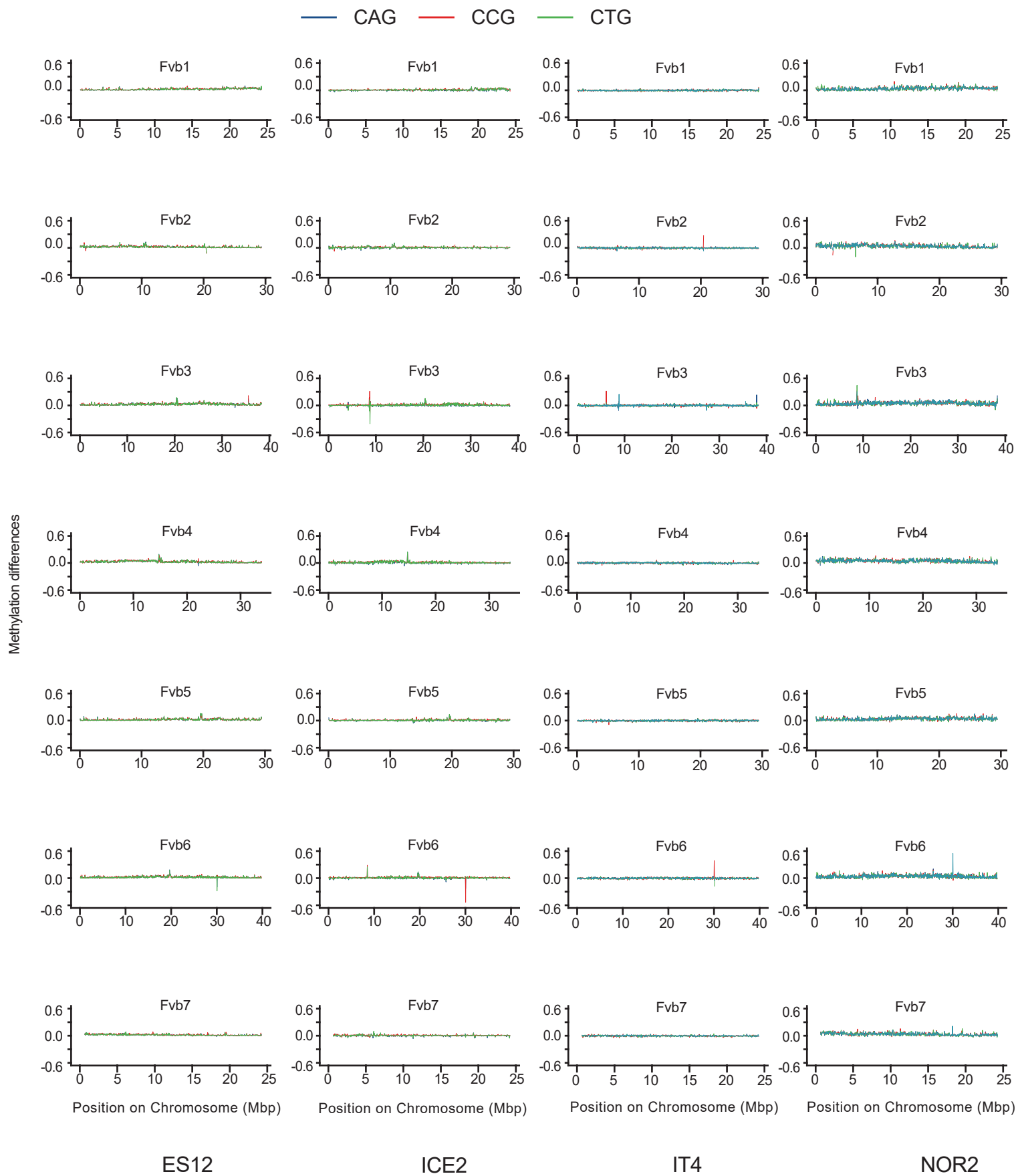

Supplement: Web_Material_uhad156 [file web_material_uhad156.zip › Supplementary Figure 12.pdf]

CAA CAC CAT  
CCA CCC CCT  
CTA CTC CTT

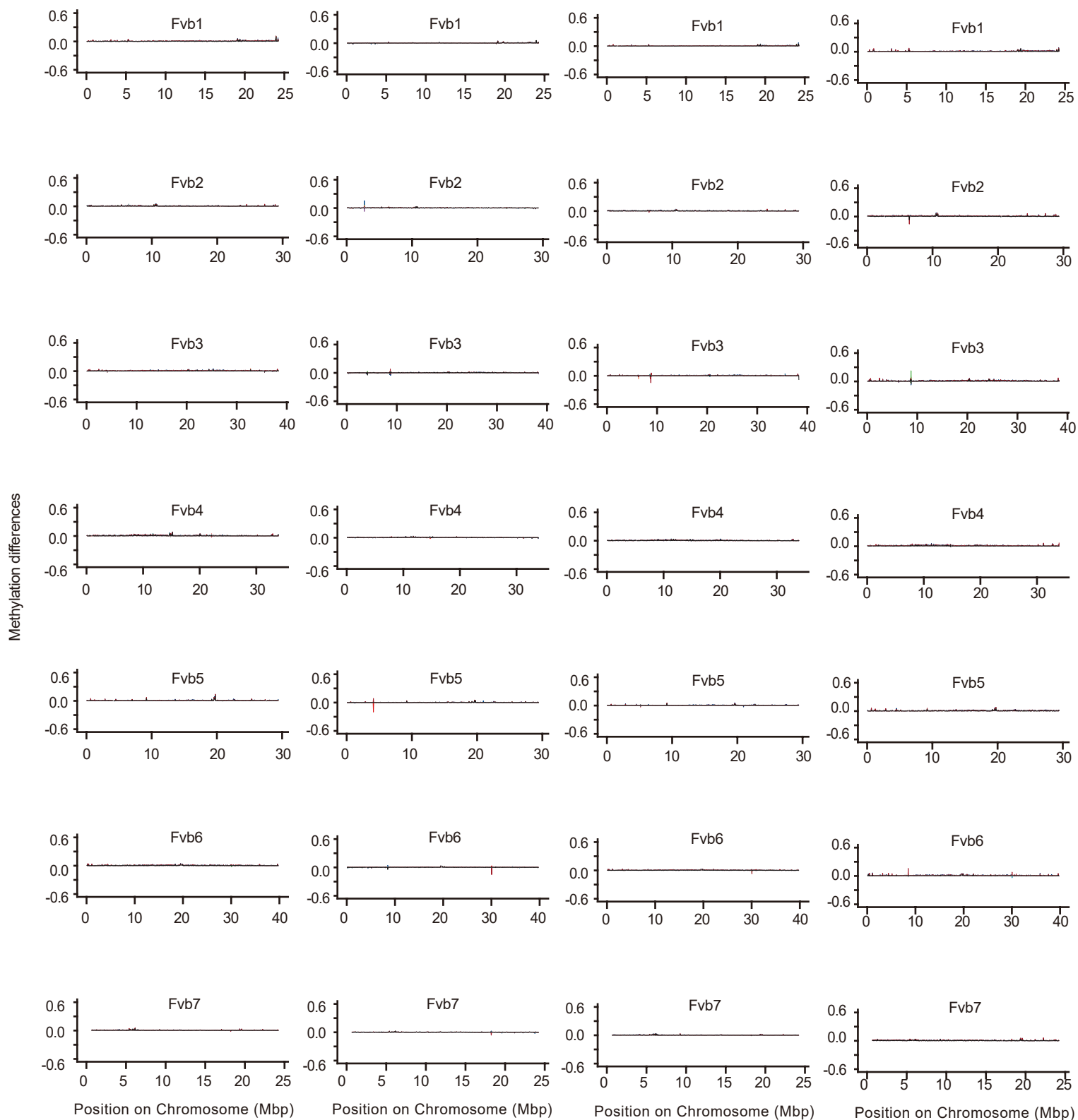

Supplement: Web_Material_uhad156 [file web_material_uhad156.zip › Supplementary Figure 13.pdf]

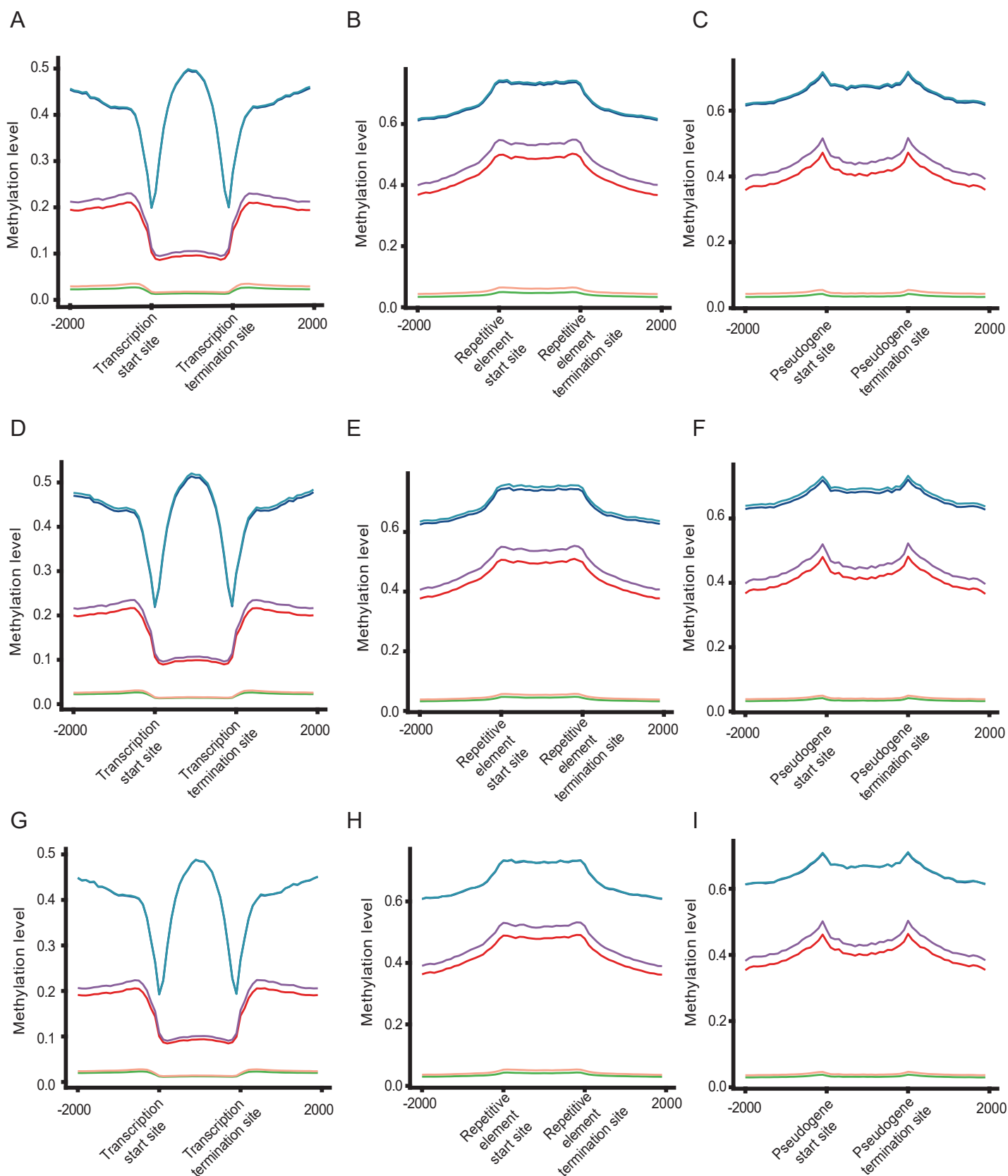

— 18°C-CGN    — 28°C-CGN  
 — 18°C-CHG    — 28°C-CHG  
 — 18°C-CHH    — 28°C-CHH

Supplement: Web_Material_uhad156 [file web_material_uhad156.zip › Supplementary Figure 14.pdf]

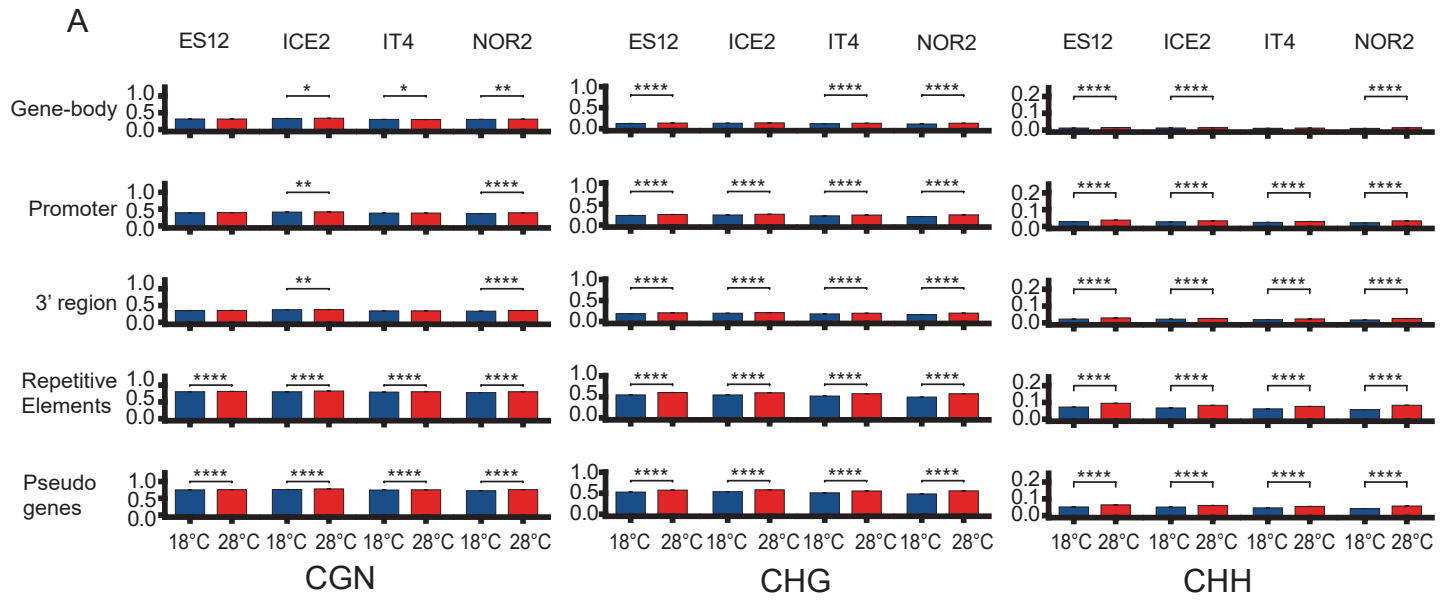

Supplement: Web_Material_uhad156 [file web_material_uhad156.zip › Supplementary Figure 15.pdf]

— ES12 — ICE2 — IT4 — NOR2

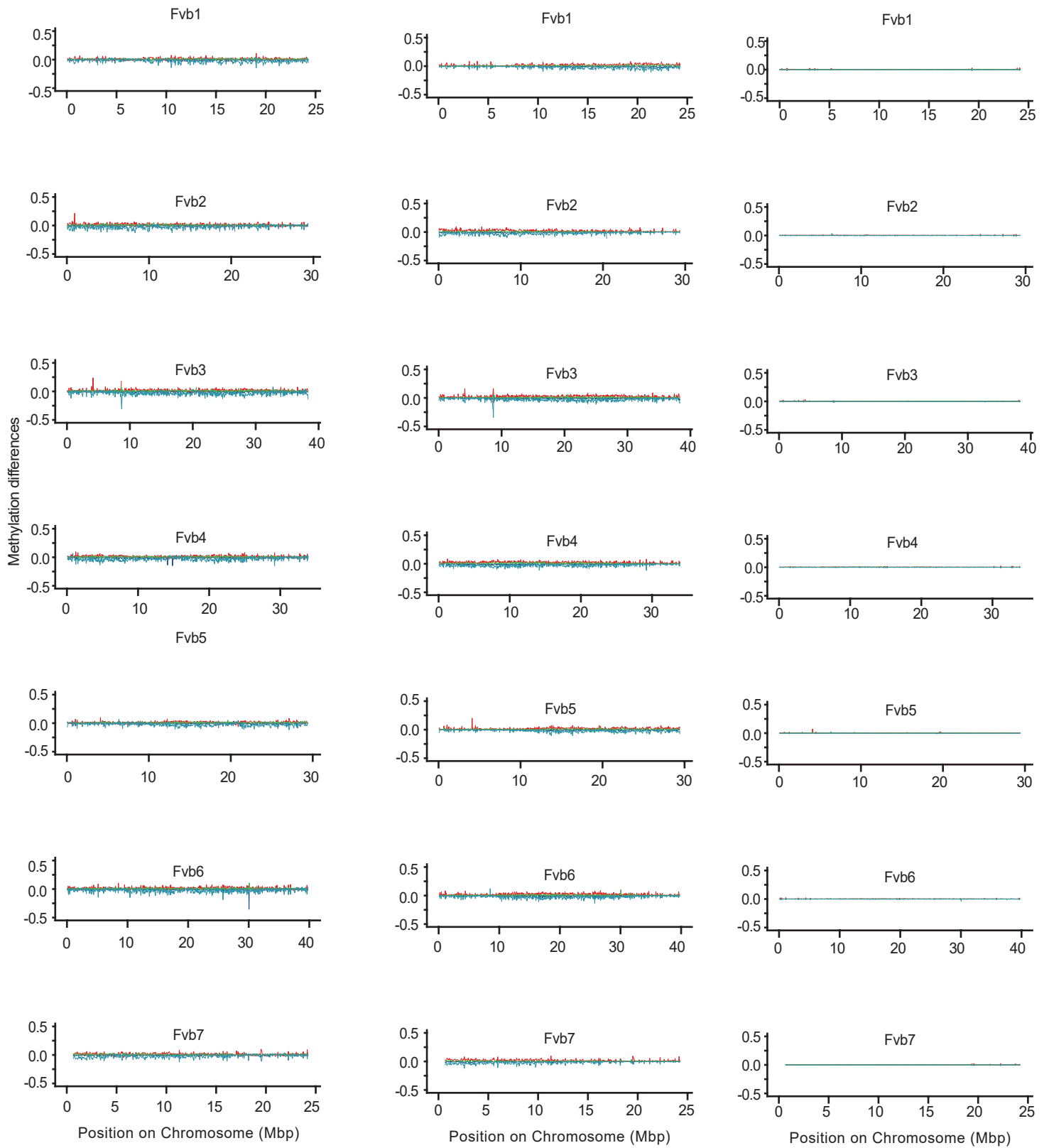

CGN

CHG

CHH

Supplement: Web_Material_uhad156 [file web_material_uhad156.zip › Supplementary Figure 17.pdf]

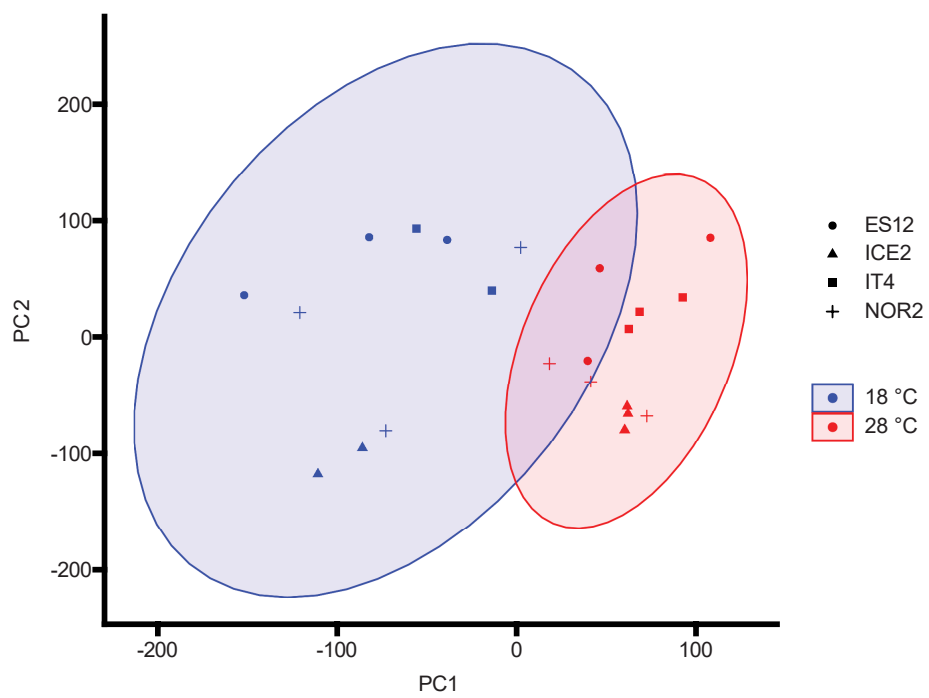

Supplement: Web_Material_uhad156 [file web_material_uhad156.zip › Supplementary Figure 19.pdf]

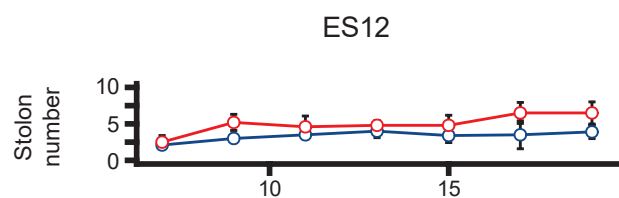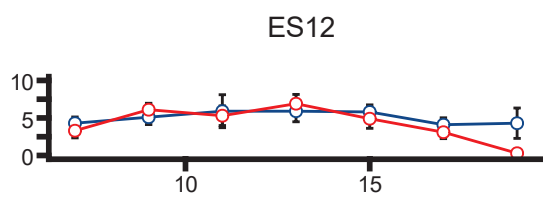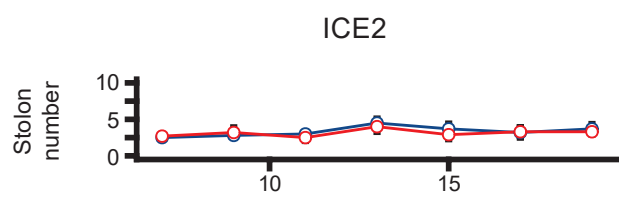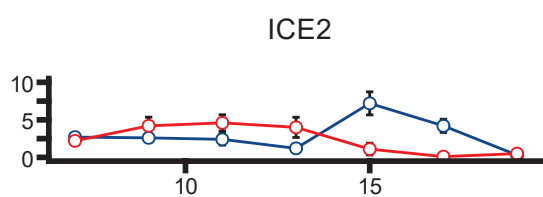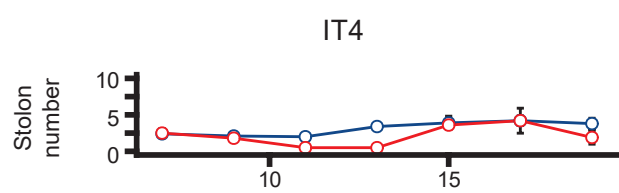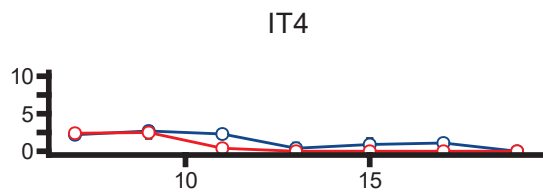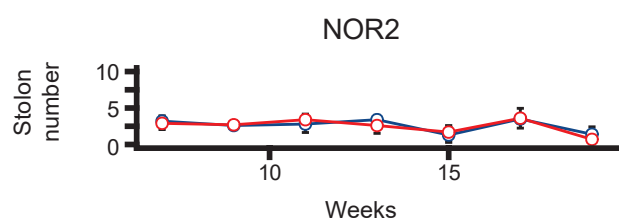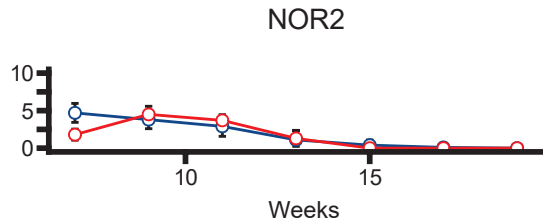

18 °C

28 °C

AS1

AS3

Supplement: Web_Material_uhad156 [file web_material_uhad156.zip › Supplementary Figure 2.pdf]

A

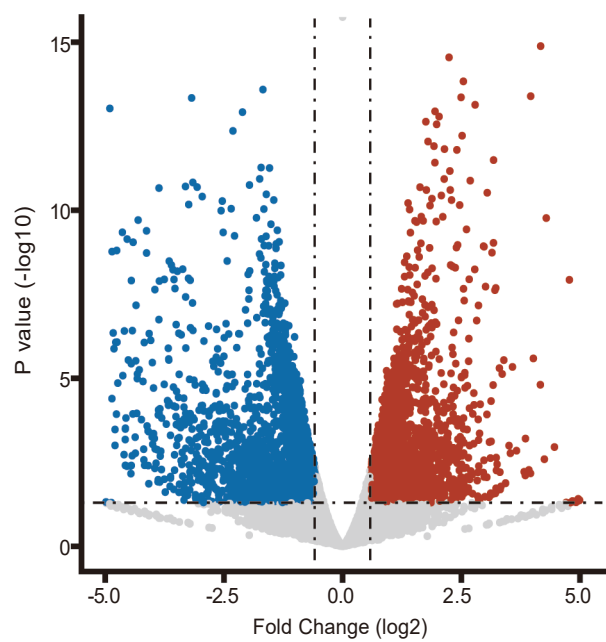

B

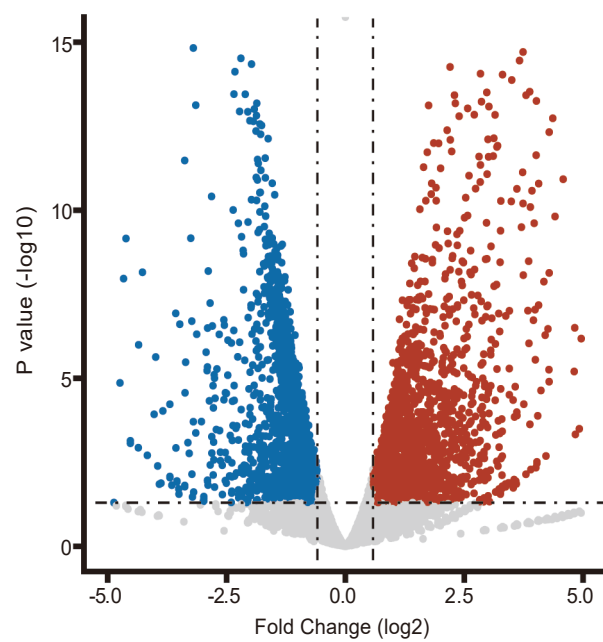

C

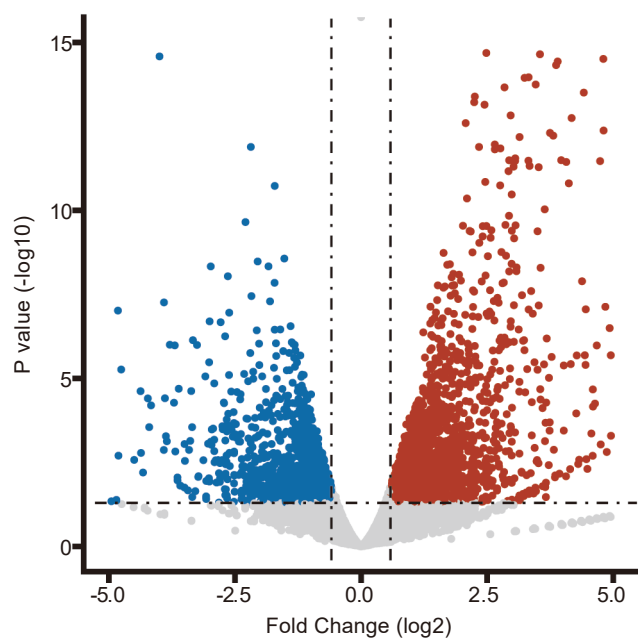

Supplement: Web_Material_uhad156 [file web_material_uhad156.zip › Supplementary Figure 20.pdf]

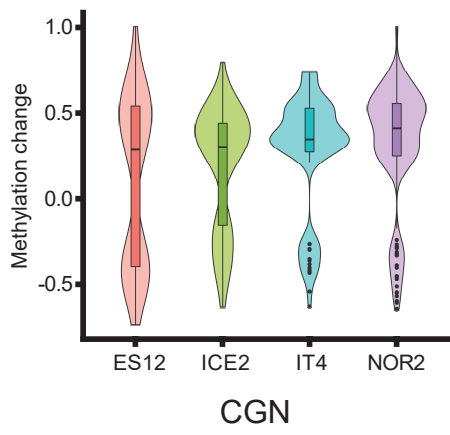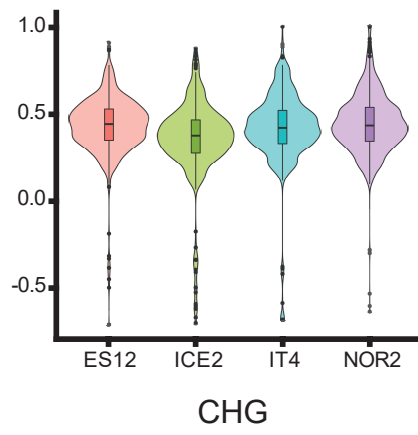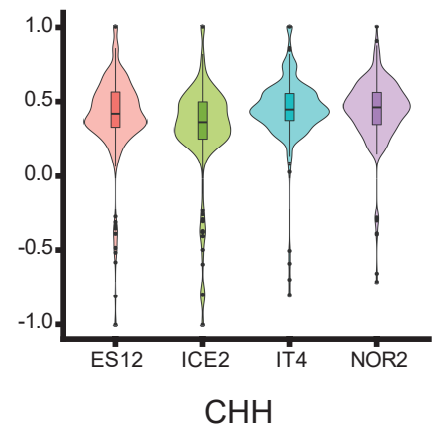

Supplement: Web_Material_uhad156 [file web_material_uhad156.zip › Supplementary Figure 25.pdf]

A

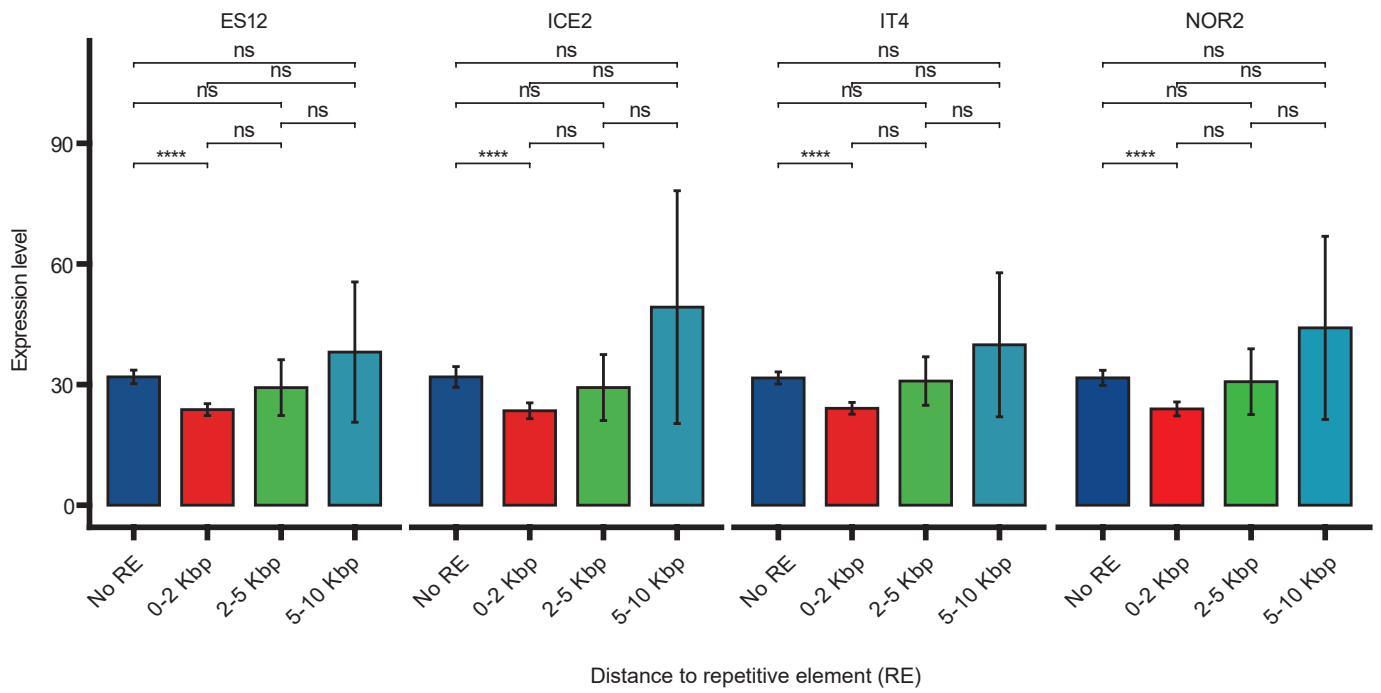

B

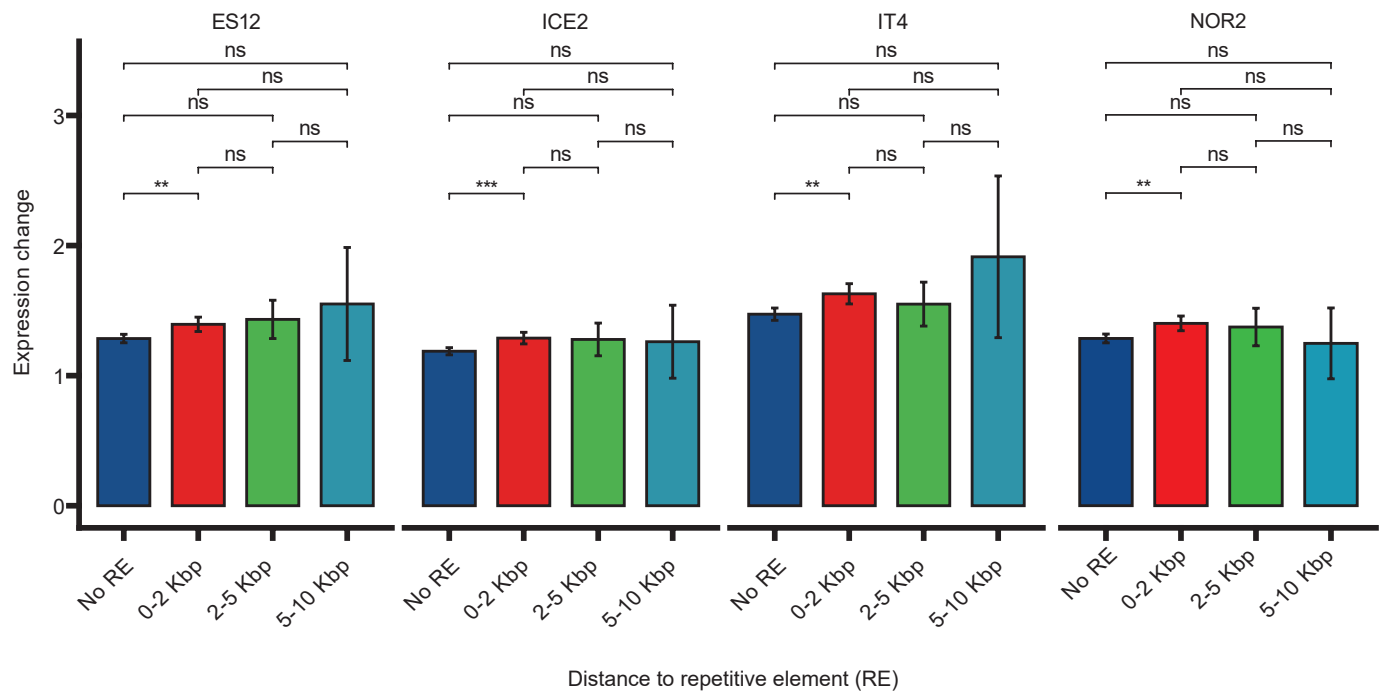

Supplement: Web_Material_uhad156 [file web_material_uhad156.zip › Supplementary Figure 26.pdf]

ES12

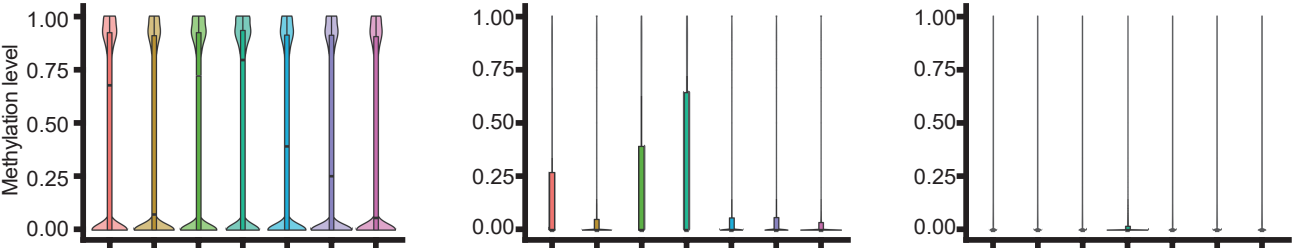

ICE2

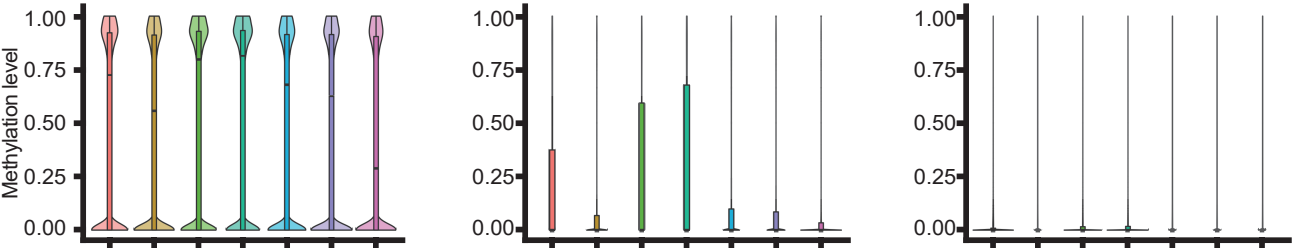

IT4

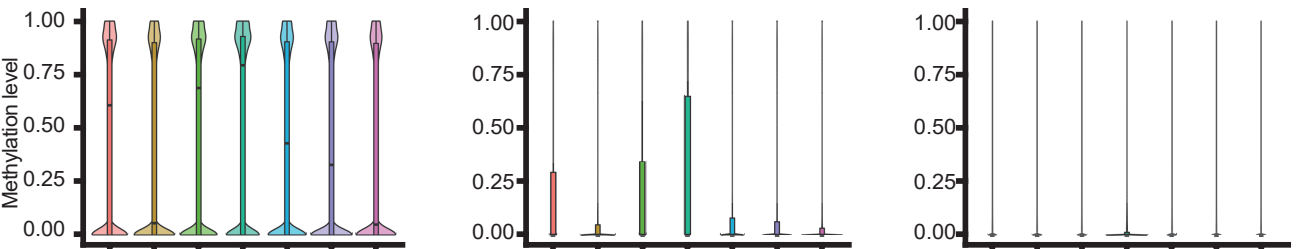

NOR2

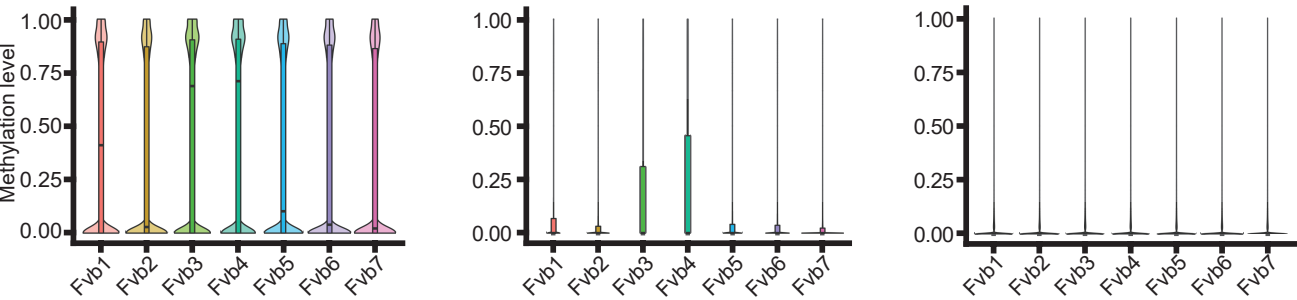

CGN

CHG

CHH

Supplement: Web_Material_uhad156 [file web_material_uhad156.zip › Supplementary Figure 3.pdf]

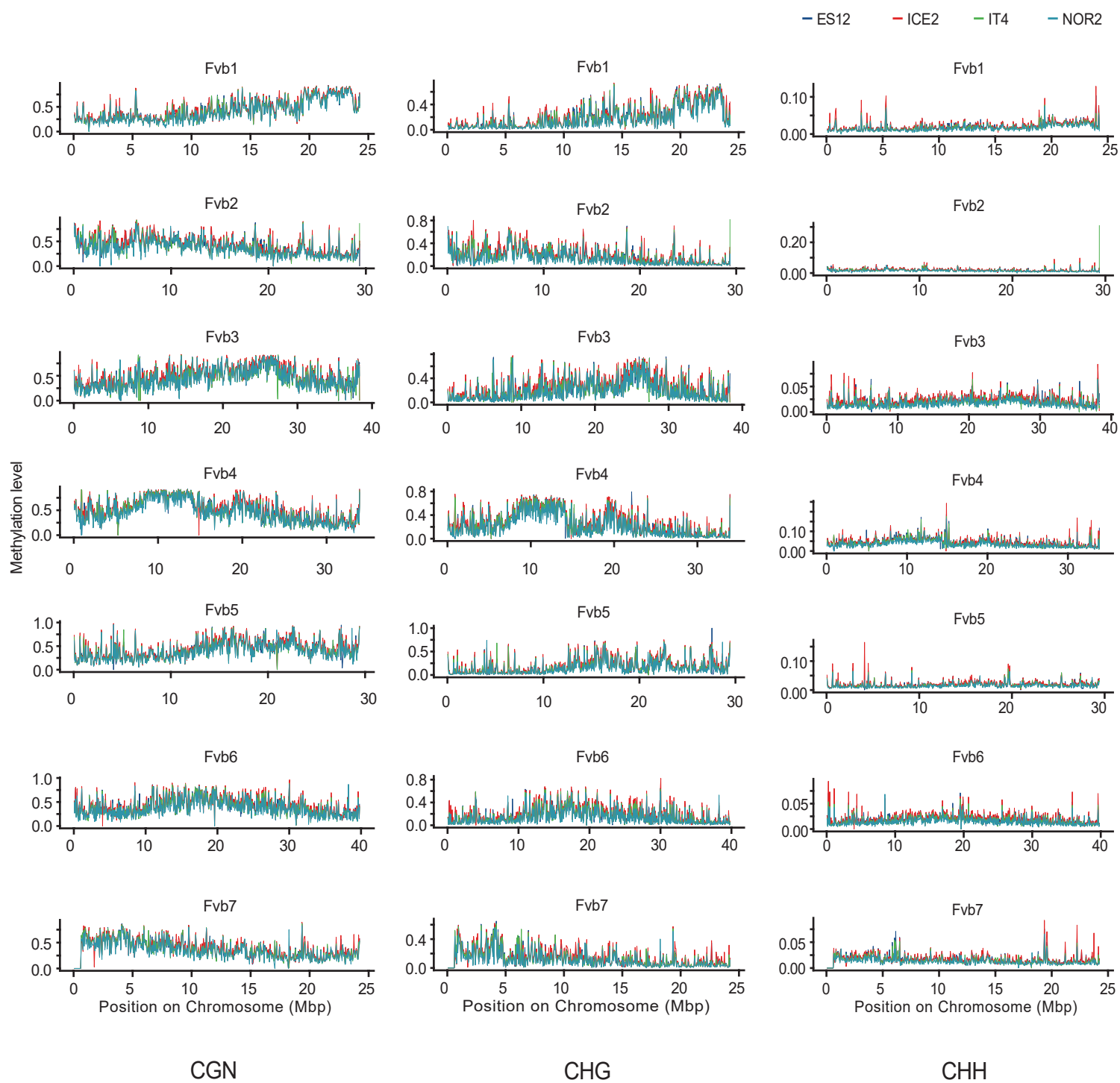

Supplement: Web_Material_uhad156 [file web_material_uhad156.zip › Supplementary Figure 4.pdf]

ICE2-ES12 IT4-ES12 NOR2-ES12

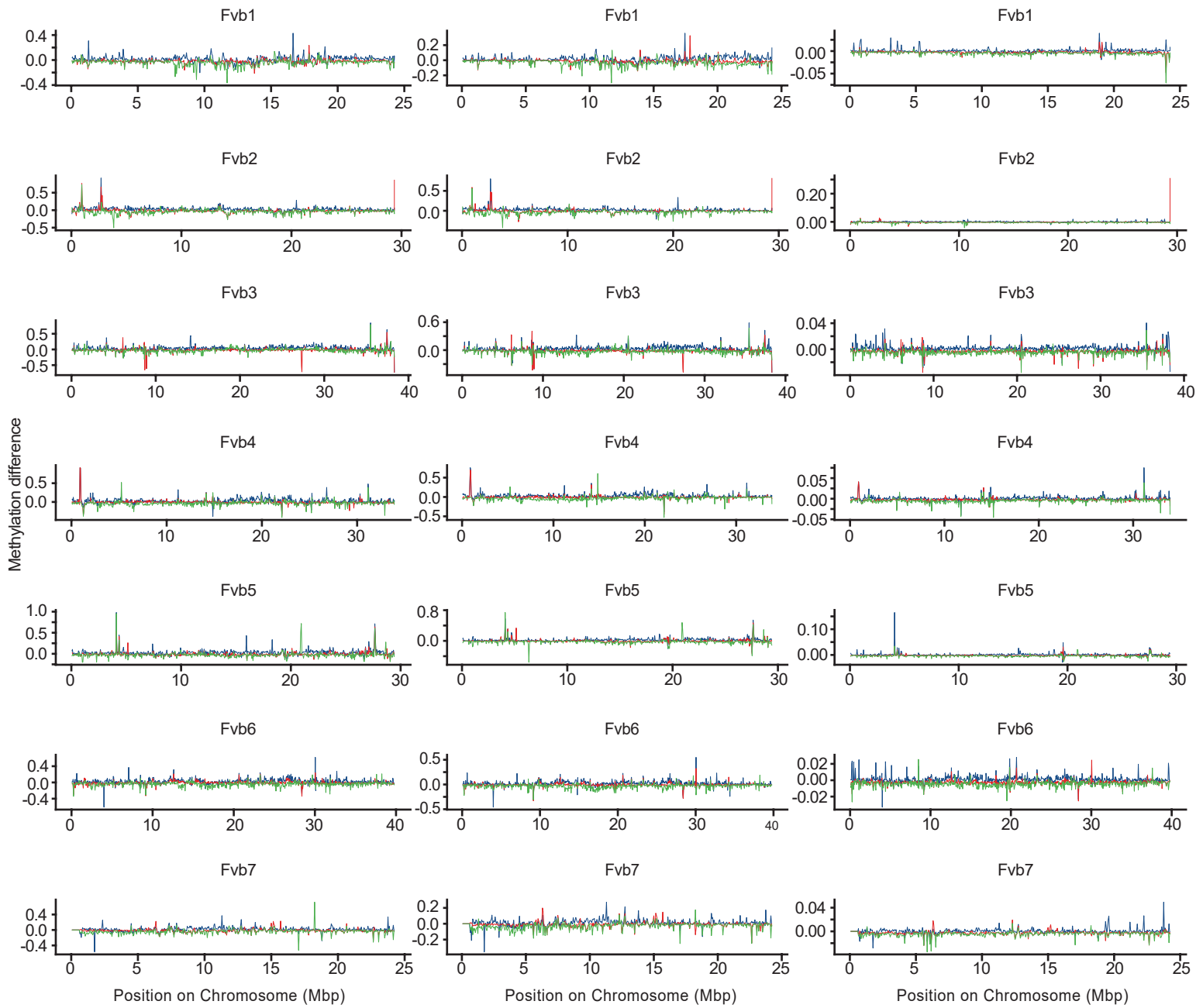

CGN

CHG

CHH

Supplement: Web_Material_uhad156 [file web_material_uhad156.zip › Supplementary Figure 5.pdf]

— CGA — CGC — CGG — CGT

Methylation level

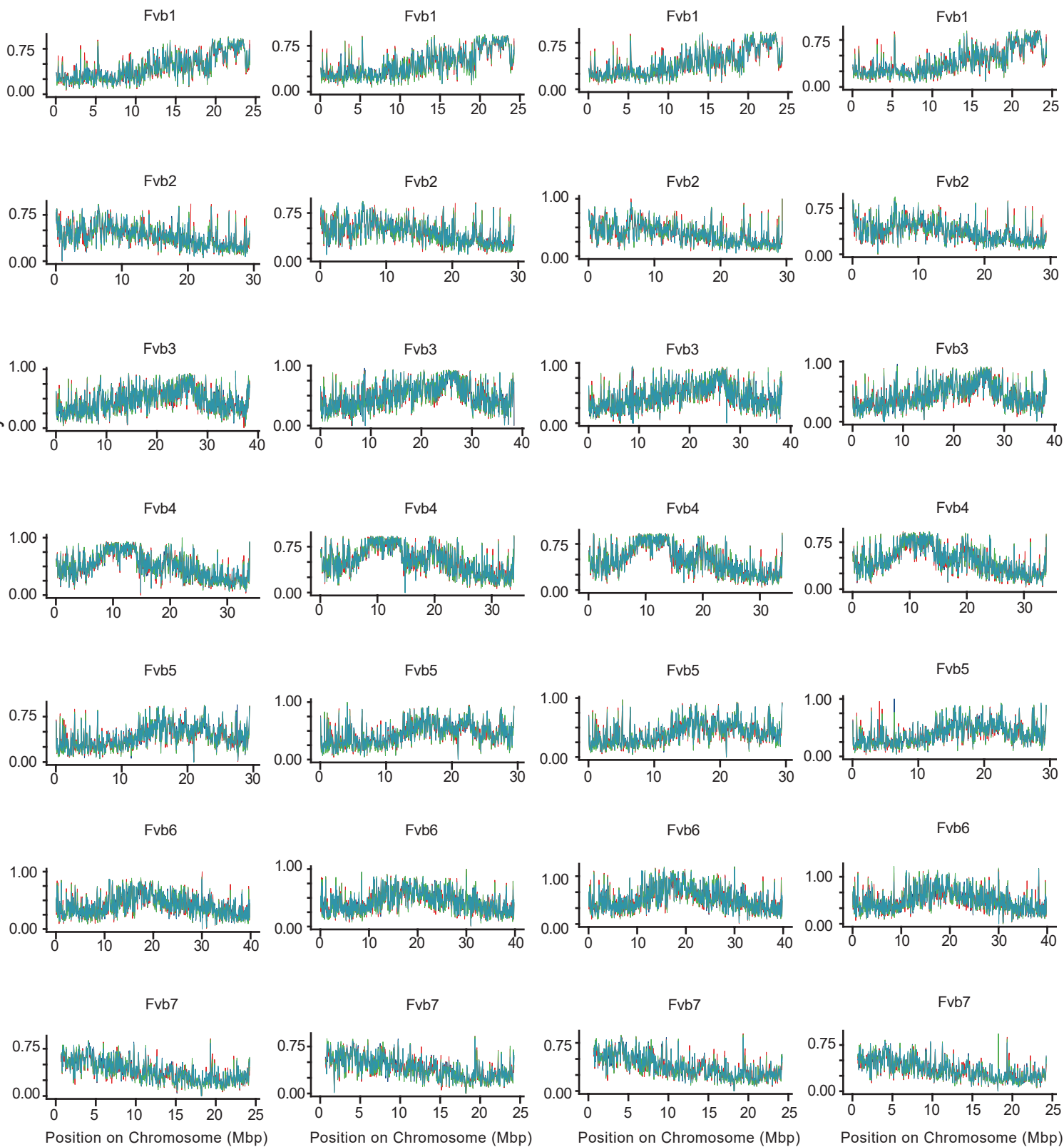

ES12

ICE2

IT4

NOR2

Supplement: Web_Material_uhad156 [file web_material_uhad156.zip › Supplementary Figure 6.pdf]

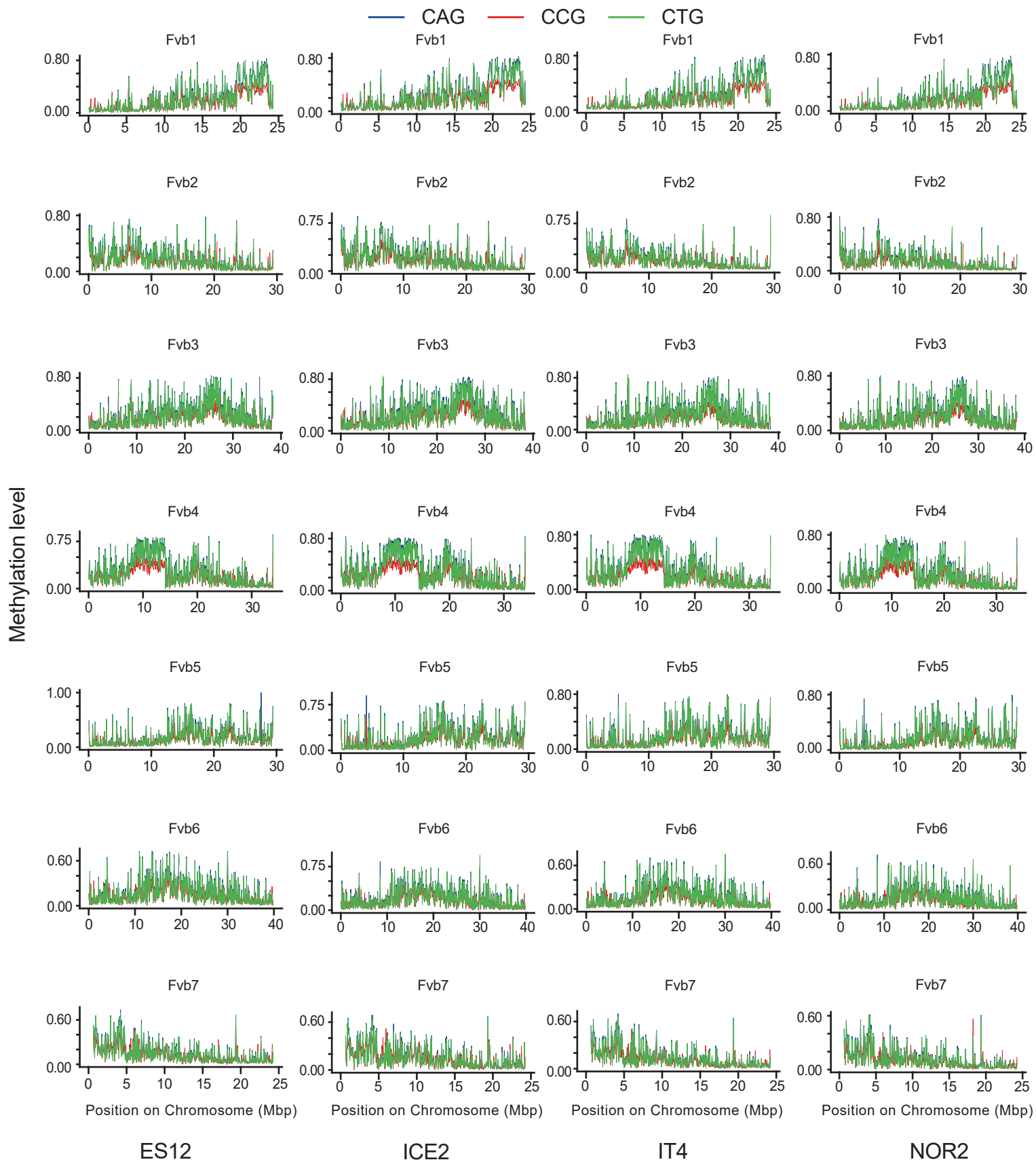

Supplement: Web_Material_uhad156 [file web_material_uhad156.zip › Supplementary Figure 7.pdf]

|       |       |       |
|-------|-------|-------|
| — CAA | — CAC | — CAT |
| — CCA | — CCC | — CCT |
| — CTA | — CTC | — CTT |

Methylation level

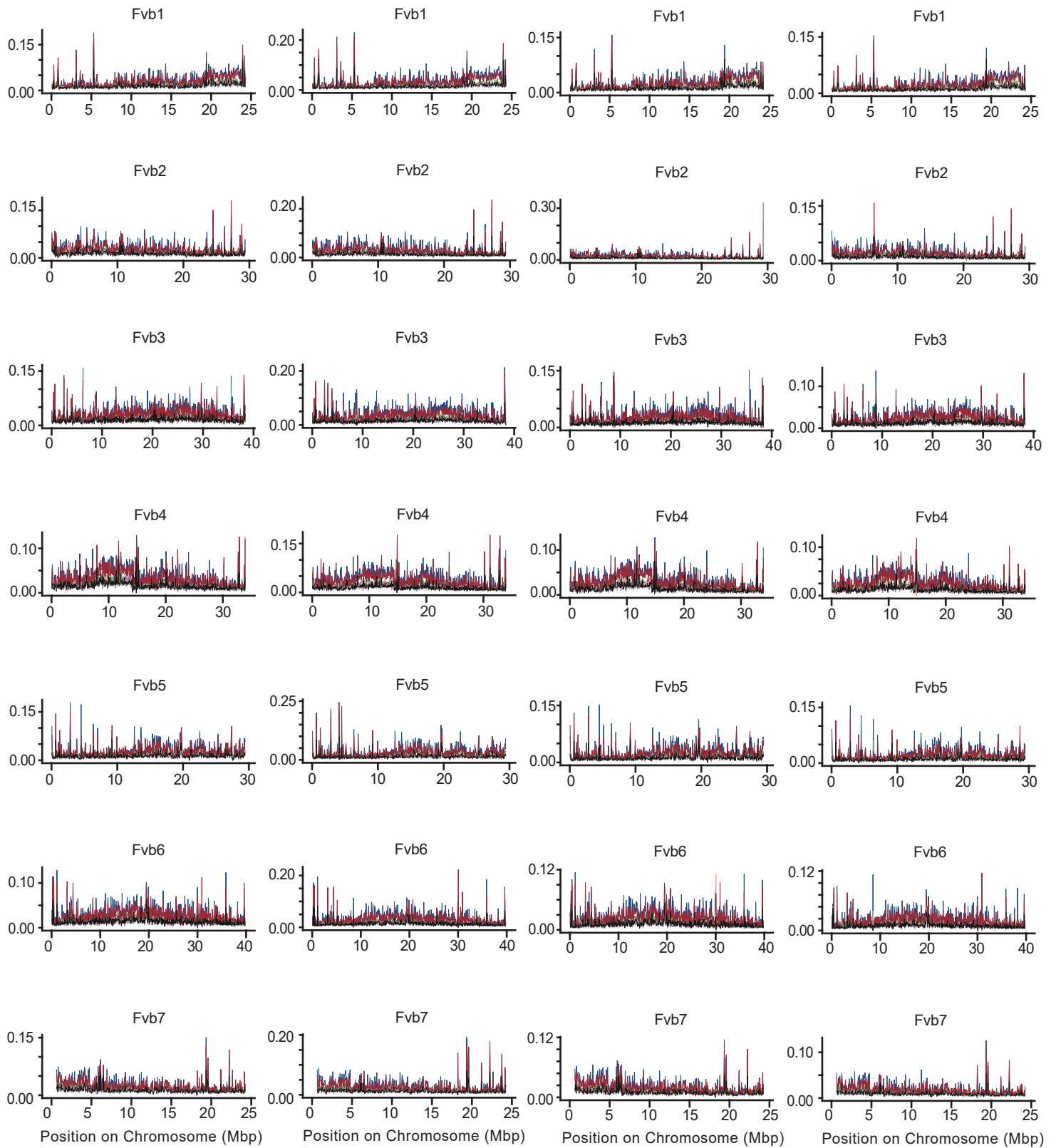

Supplement: Web_Material_uhad156 [file web_material_uhad156.zip › Supplementary Figure 8.pdf]

D

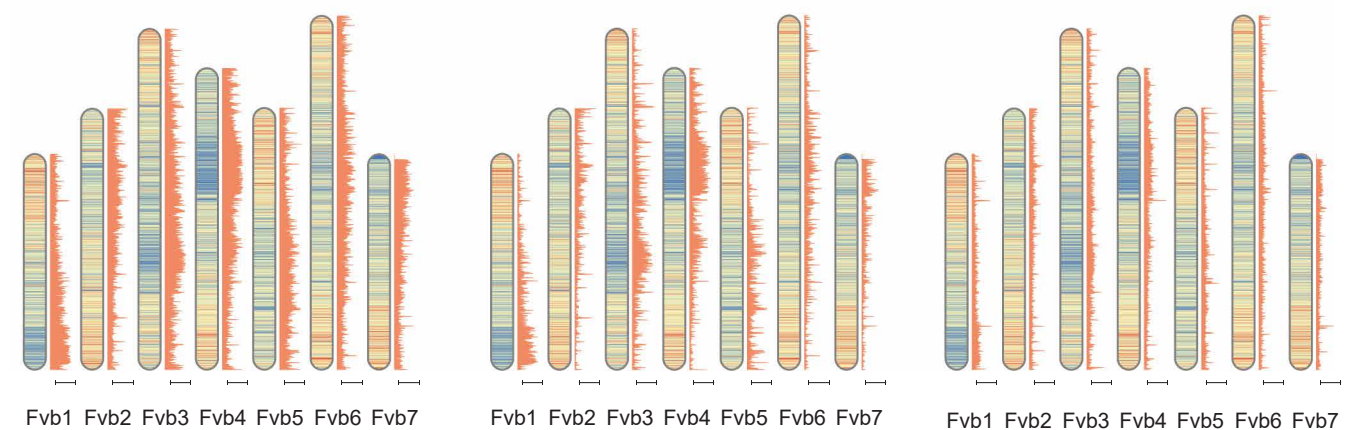

CHH

Supplement: Web_Material_uhad156 [file web_material_uhad156.zip › Supplementary Figure 9.pdf]
